# Diversity analysis of genes encoding Mfa1 fimbrial components in *Porphyromonas gingivalis* strains

**DOI:** 10.1371/journal.pone.0255111

**Published:** 2021-07-26

**Authors:** Kotaro Sakae, Keiji Nagano, Miyuna Furuhashi, Yoshiaki Hasegawa

**Affiliations:** 1 Department of Microbiology, School of Dentistry, Aichi Gakuin University, Nagoya, Japan; 2 Department of Endodontics, School of Dentistry, Aichi Gakuin University, Nagoya, Japan; 3 Division of Microbiology, Department of Oral Biology, School of Dentistry, Health Sciences University of Hokkaido, Hokkaido, Japan; 4 Department of Pediatric Dentistry, School of Dentistry, Aichi-Gakuin University, Nagoya, Japan; Medical University of South Carolina, UNITED STATES

## Abstract

*Porphyromonas gingivalis*, a gram-negative anaerobic bacterium, is associated with the development of periodontal disease. The genetic diversity in virulence factors, such as adhesive fimbriae, among its strains affects the bacterial pathogenicity. *P*. *gingivalis* generally expresses two distinct types of fimbriae, FimA and Mfa1. Although the genetic diversity of *fimA*, encoding the major FimA fimbrilin protein, has been characterized, the genes encoding the Mfa1 fimbrial components, including the Mfa1 to Mfa5 proteins, have not been fully studied. We, therefore, analyzed their genotypes in 12 uncharacterized and 62 known strains of *P*. *gingivalis* (74 strains in total). The *mfa1* genotype was primarily classified into two genotypes, 53 and 70. Additionally, we found that genotype 70 could be further divided into two subtypes (70A and 70B). The diversity of *mfa2* to *mfa4* was consistent with the *mfa1* genotype, although no subtype in genotype 70 was observed. Protein structure modeling showed high homology between the genotypes in Mfa1 to Mfa4. The *mfa5* gene was classified into five genotypes (A to E) independent of other genotypes. Moreover, genotype A was further divided into two subtypes (A1 and A2). Surprisingly, some strains had two *mfa5* genes, and the 2^nd^ *mfa5* exclusively occurred in genotype E. The Mfa5 protein in all genotypes showed a homologous C-terminal half, including the conserved C-terminal domain recognized by the type IX secretion system. Furthermore, the von Willebrand factor domain at the N-terminal was detected only in genotypes A to C. The *mfa1* genotypes partially correlated with the *ragA* and *ragB* genotypes (located immediately downstream of the *mfa* gene cluster) but not with the *fimA* genotypes.

## Introduction

*Porphyromonas gingivalis*, a gram-negative anaerobic bacterium, is associated with the development and progression of periodontal disease [1]. This bacterium is a keystone pathogen that has a crucial influence on a microbial niche [2, 3]. As such, *P*. *gingivalis* leads the periodontal microbiota to dysbiosis, an imbalanced state of the microbiota, which then evokes inflammation in gingival tissues, although the bacterium is only present in a small quantity [2, 3].

*P*. *gingivalis* expresses various virulence factors, most notably fimbria, which functions to form a multi-species biofilm and colonizes the periodontal tissue [4]. At least two distinct types of fimbriae are expressed by the bacterium, namely, the FimA and Mfa1 fimbriae (Yoshimura, 2009 #1402). Other virulence factors also include gingipain, a trypsin-like protease, which causes tissue damage in the host [5]. Furthermore, capsules and lipopolysaccharides are known to facilitate immune evasion and inflammation induction, respectively [5]. However, *P*. *gingivalis* is also present in healthy subjects and shows divergent properties in pathology, indicating that there is genetic diversity in its virulence factors [6–8].

The *fimA* gene was the first to be identified as responsible for the diversity in *P*. *gingivalis* [9–11]. The *fimA* gene encodes a major fimbrilin protein (FimA), which polymerizes into a filament of FimA fimbriae [12]. The *fimA* gene has been classified into six genotypes (I to V, and Ib) based on either PCR using genotype-specific primers, or on the presence or absence of restriction enzyme cleavage [8]. However, genotype Ib is now unified into genotype I because of their similar antigenicity and overall DNA sequences [13, 14]. Studies from many countries have reported that genotypes II and IV are predominantly detected in patients with severe periodontitis, whereas genotype I is prevalent in healthy or mild periodontitis subjects [8, 15, 16]. However, genotype I is also reportedly detected at a high frequency in severe periodontitis [17, 18]. Moreover, a previous study showed no association between the *fimA* genotype and bacterial pathogenicity [19]. The discrepancies between these results indicate that the pathogenic diversity in *P*. *gingivalis* cannot be explained by the *fimA* genotype alone.

We previously found two genotypes in the *mfa1* gene, which encodes the major fimbrilin of Mfa1 fimbriae, and called them 53 (kDa) and 70 (kDa) genotypes based on the apparent molecular weight of the Mfa1 proteins encoded by the gene [20, 21]. The DNA and amino acid sequences of the representative strains of genotypes 53 (strain Ando) and 70 (strain ATCC 33277) showed only 52.6% and 38.1% similarity, respectively. Additionally, these two genotypes exhibit differential antigenicity [20]. However, we did not detect any relationship between the *mfa1* genotype and the severity of periodontal disease [21].

**Fig 1:**
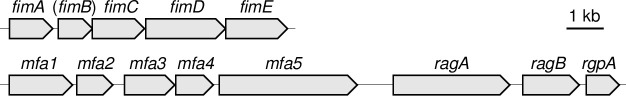
Gene map of the *fim*, *mfa*, and *rag* gene clusters of a type strain (ATCC 33277) of *P*. *gingivalis*. There are distances between the *fim* gene cluster (*fimA* to *fimE*), as well as *mfa* (*mfa1* to *mfa5*) and *rag* (*ragA* and *ragB*) gene clusters. The *fimB* gene is placed in parentheses because there is a nonsense mutation in ATCC 33277. Arrows depict gene directions from 5՛ to 3՛.

The FimA and Mfa1 proteins polymerize to form a filamentous structure by a similar mechanism [22]; *via* proteolytic processing by a signal peptidase and gingipain to yield the mature forms. Then, the C-terminal donor strands of the incoming monomer extend and bind to the hydrophobic groove of the prior monomer [23, 24]. This is known as the donor-strand exchange mechanism in the assembly of *Escherichia coli* fimbriae [25], but unlike in *P*. *gingivalis*, chaperone and usher proteins are absent, whereas digestion with protease (gingipain) is involved in maturation [22]. This proteinase-mediated donor-strand exchange mechanism, currently seen only in the class Bacteroidia, is called type V fimbriae [23]. FimA and Mfa1 fimbriae have four additional accessory proteins, FimB to FimE, and Mfa2 to Mfa5, respectively [4]. Genomic analysis of the ATCC 33277 type strain revealed that the genes encoding the FimA and Mfa1 fimbriae-associated proteins form respective clusters: *fimB* to *fimE* arrange immediately downstream of *fimA*, while *mfa1* to *mfa5* sequentially arrange (Fig 1). FimB and Mfa2 also show similar biogenesis and function [23, 26–28]. During maturation, they are digested by a signal peptidase, but not by gingipain. They localize at the base of the respective fimbriae and function to anchor the fimbrial filament to the bacterial body [26, 27]. Additionally, integration of FimB and Mfa2 into the respective fimbriae terminates fimbrial elongation [26, 27]. During maturation of FimC, FimD, FimE, Mfa3, and Mfa4, digestion with gingipain is necessary [23, 28–32]. They function as adhesins and facilitate fimbrial assembly [23, 28–33]. Unlike the other proteins described above, Mfa5 first translocates to the periplasmic space *via* a signal peptide and then to the outer membrane by the type IX secretion system (T9SS) [32]. T9SS secretes a bacterial protein that is uniquely found in the phylum Bacteroidetes [34]. Proteins recognized by the T9SS have a characteristic motif called the C-terminal domain (CTD) [35], which is found in Mfa5 of ATCC 33277 [32]. Recently, X-ray crystallography revealed that Mfa5 contained one von Willebrand factor (VWF) domain and two Ig-like domains in the N-terminal half [36]. It also showed that a loop structure called ARM2 is adjunct to the VWF domain. Furthermore, the observed formation of isopeptide bonds (between Lys^111^ and Asn^518^ of Mfa5 in ATCC 33277) reportedly only occurs in gram-positive bacteria such as streptococci [36].

The *rag* gene cluster contains the *ragA* and *ragB* genes immediately downstream of the *mfa* gene cluster in ATCC 33277 (Fig 1). They encode proteins localized in the outer and inner membranes, respectively, and associate with each other to function in nutrient uptake [37–39]. It has also been shown to stimulate innate immunity and induce inflammation [40]. There are four genetic polymorphisms in *ragA* and *ragB*, and, therefore, four potential associations between the genotypes and bacterial pathogenicity [37].

Previously, we did not determine the *mfa1* genotype in 12 of the 84 strains of *P*. *gingivalis* in western blot and PCR analyses [20]. This suggested the existence of a novel *mfa1* genotype. Therefore, in the present study we analyzed the draft genome of the 12 strains by next-generation sequencing (NGS) to define their *mfa1* genotypes. We also analyzed the genomes of the additional 62 strains of *P*. *gingivalis* published in the expanded Human Oral Microbiome Database (eHOMD) (http://www.homd.org/) to examine the genomic diversity in the *mfa* gene cluster. Lastly, we analyzed the correlation of genetic diversity in the *mfa* gene cluster with those in the *fim* and *rag* gene clusters.

## Materials and methods

### *P*. *gingivalis* strains

We analyzed the genomic data of 12 unsequenced strains (222, 1436, 1439, B42, B158, D83T3, EM3, JKG9, JKG10, Kyudai-3, Kyudai-4, and TV14) [13] as described below. We also used genomic information from 62 sequenced strains (381, 11A, 13_1, 15_9, 3_3, 381OKJP, 3A1, 7BTORR, 84_3, A7436, A7A1-28, AFR5B1, AJW4, Ando, ATCC 33277, ATCC 49417, CP3, F0185, F0566, F0568, F0569, F0570, H3, HG66, JCVI SC001, KCOM 2796, KCOM 2797, KCOM 2798, KCOM 2799, KCOM 2800, KCOM 2801, KCOM 2802, KCOM 2803, KCOM 2804, KCOM 2805, KCOM 3001, KCOM 3131, MP4-504, SJD11, SJD12, SJD2, SJD4, SJD5, SU60, TDC 60, UBA8864, W4087, W50, W83, WW2096, WW2842, WW2866, WW2881, WW2885, WW2903, WW2931, WW2952, WW3039, WW3040, WW3102, WW5019, and WW5127) published on the eHOMD website.

### NGS analysis

We analyzed the genome sequence of 12 *P*. *gingivalis* strains whose *mfa1* genotypes have not been determined as described above [20] (Table 1). The strains were maintained on Brucella HK agar (Kyokuto Pharmaceutical Industrial, Tokyo, Japan) supplemented with 5% rabbit blood, defibrinated at 37°C under anaerobic conditions. A black pigmented colony was inoculated and cultivated in the GAM broth, Modified (Nissui Pharmaceutical, Tokyo, Japan) to collect the bacterial cells. Chromosomal DNA was extracted using the Wizard Genomic DNA Purification Kit (Promega Corporation, Madison, WI, USA) and subjected to NGS analysis. The draft genome sequences were analyzed by Filgen (Nagoya, Japan), and 150-bp paired-end sequences generated by NovaSeq (Illumina, San Diego, CA, USA) were *de novo* assembled using ABySS version 2.2.4 [41] using the bioinformatics software OmicsBox (BioBam Bioinformatics S.L., Valencia, Spain). The optimal *k*-mer values were determined using the total length of the assembled sequence as an index.

**Table 1:** Basic information of genome-sequencing of 12 strains in this study.

Strain	Optimal	Total	Contig	*N*_*50*_	Accession number
	*k*-mer	length	number		
222	123	2,333,098	90	49,404	JAEMBR000000000
1436	127	2,331,789	110	34,325	JAEMBQ000000000
1439	127	2,253,658	87	43,390	JAEMBL000000000
B42	127	2,282,399	89	42,113	JAEMBK000000000
B158	127	2,265,994	85	51,392	JAEMBJ000000000
D83T3	127	2,440,602	134	33,563	JAEMBP000000000
EM3	123	2,332,868	115	33,606	JAEMBI000000000
JKG9	127	2,382,938	97	36,654	JAEMBH000000000
JKG10	127	2,366,674	112	37,506	JAEMBG000000000
Kyudai-3	123	2,424,555	116	36,180	JAEMBO000000000
Kyudai-4	127	2,247,019	107	44,629	JAEMBN000000000
TV14	127	2,395,514	113	53,426	JAEMBM000000000

### Bioinformatics

Bioinformatics analysis was performed using the programs listed in Table 2. Detection of *mfa*, *fim*, and *rag* gene clusters was performed using BLAST on the web. When the 12 strains sequenced in this study were analyzed, we used BLAST with the “Align two or more sequences” enabled. The assembly data including all contigs were entered in a box for “query sequence,” and a probe sequence was entered in “subject sequence.” Then, the contig containing the probe sequence was obtained for further analysis. When the strains published in the eHOMD site were analyzed, the target genes were detected using a tool of “Genome Viewer” in the eHOMD site, and the contig containing the target sequence was obtained. The eHOMD shows open reading frames (ORFs) and amino acid sequences deduced from the ORFs. However, we found different ORFs between the strains, although their DNA sequences were almost identical. We modified the ORFs to align the start codon among the strains and then analyzed the DNA sequences. We determined the bacterial species through the DNA sequences of 16S rRNA and multilocus sequence typing analysis (MLST) of seven genes of *P*. *gingivalis* including *pepO*, *gpdxJ*, *hagB*, *recA*, *mcmA*, *pga*, and *ftsQ* in the PubMLST site [7]. A phylogenetic tree was constructed using TreeView X through a multiple sequence alignment analysis using ClustalΩ version 1.2.2. The genotype was classified based on the phylogenetic distance and cluster formation; when the phylogenetic distance between the genes was less than 0.1, they were classified into a single genotype. However, even when the phylogenetic distance was less than 0.1, genes were classified into different genotype considering the genotype of the adjacent genes. Furthermore, if there were multiple distinct clusters within a genotype, they were classified as subtypes.

**Table 2:** Program and web site of bioinformatics used in this study.

Name	URL	Description
HOMD	http://www.homd.org/	Database of oral microbiome
BLAST	https://blast.ncbi.nlm.nih.gov/Blast.cgi	Homology search for DNA/amino acid sequences
PubMLST	https://pubmlst.org/	Identification of bacterial species by multilocus sequence typing
Clustal Omega (Ω)	https://www.ebi.ac.uk/Tools/msa/clustalo/	Alignment and phylogenic analyses of multiple DNA/amino acid sequences
TreeView X	http://taxonomy.zoology.gla.ac.uk/rod/treeview.html	Drawing program of a phylogenic tree from the result of ClustalΩ analysis
SignalP 5.0	http://www.cbs.dtu.dk/services/SignalP-5.0/	Prediction of the signal peptides in proteins
SWISS-MODEL	https://swissmodel.expasy.org/	Protein structure homology-modeling
Protein Data Bank	https://www.rcsb.org/pages/publications	Database of 3D structure of proteins

Additionally, the genotypes of *fimA*, *ragA*, and *ragB* were named according to the same terminology as those in previous papers: *fimA* was classified as genotype I to V [8], whereas *ragA* and *ragB* were classified into genotypes 1 to 4 (or *ragA-1*/*ragB-1* to *ragA-4*/*ragB-4*) [42]. A signal peptide was predicted using SignalP 5.0. SWISS-MODEL analysis was used for protein structure homology modeling based on the X-ray crystal structures deposited in the Protein Data Bank (PDB). The X-ray crystal structures of the mature forms of Mfa1 [28, 43], Mfa2 [28, 43], Mfa3 [28, 29], and Mfa4 [23, 30] proteins, and the N-terminal portion of the Mfa5 protein [36] have been published at the PDB site previously. All structural data were based on the DNA sequence of ATCC 33277.

## Results

### Draft genome sequencing and detection of the *mfa1* gene in the 12 unsequenced strains

NGS analysis produced a draft genome sequence of the 12 strains whose *mfa1* genotypes were not determined previously [20]. The results are summarized in Table 1 with GenBank accession numbers. All strains were assembled into approximately 100 contigs, with a total length comparable to the genome sizes of strains ATCC 33277 (2,354,886 bp [44]) and W83 (2,343,476 bp [45]). They also showed high-quality values of *N*_*50*_ with more than 33,000. We confirmed the presence of *P*. *gingivalis* through the DNA sequences of 16S rRNA and MLST analysis with 99.5 to 100% identity.

BLAST searches of the *mfa* gene cluster from the draft genomes of the 12 strains are shown in Table 3. The *mfa1* gene was detected in 11 strains, but not in strain 222. Strain 222 showed only the latter half of *the mfa5*, and *traA* and *traB* genes encoding conjugative transposon proteins upstream of the truncated *mfa5* gene. A possible nonsense mutation in *mfa1* was detected in B158, Kyudai-4, and TV14, which are unlikely to express the Mfa1 protein. In D83T3 and Kyudai-3, although the entire *mfa1* gene exists, the insertion sequence (IS) could be seen immediately downstream of it, and the contig was different from *mfa2* and beyond. In JKG10, the first half of the *mfa1* gene was not detected because the gene was located at the end of a contig, and it was therefore not possible to determine whether the full length of the gene existed. The *rag* and *fim* gene clusters were detected in all the strains, including 222.

**Table 3:** Genotypes in the *mfa*, *rag*, and *fim* gene clusters.

	*mfa1*	*mfa2*	*mfa3*	*mfa4*	*mfa5-1*	*mfa5-2*	*ragA*	*ragB*	*fimA*	*fimB*	*fimC*	*fimD*	*fimE*
84_3	53	53	53	53	A1		1	1	I	-	A	A	A
7BTORR	53	53	53	53	A1		2	2	II	-	A	A	A
WW2866	53	53	53	53	A1		3	3	Ⅱ	-	A	A	A
A7A1-28	53	53	53	53	A1 (M)		3	3	Ⅱ	-	A	A	A
KCOM 3131	53	53	53	53	A2	E	1	1	Ⅰ	-	A	A	A
KCOM 2801	53	53	53	53	A2	E	1	1	I	-	A	A	A
WW2931	53	53	53	53	A2	E	1	1	V	-	B	B	B
WW2903	53	53	53	53	A2	E	2	2	II	-	A	A	A
AJW4	53	53	53	53	A2	E	3	3	Ⅱ	-	A	A	A
KCOM 2796	53	53	53	53	A2	E	3	3	II	-	A	A	A
SJD2	53	53	53	53	A2	E	3	3	I	-	B	B	B
KCOM 2798	53	53	53	53	A2	E (M)	1	1	II	-	A	A	A
AFR5B1	53	53	53	53	Ns	E	2	2	Ⅰ	-	A	A	A
11A	53	53	53	53	Ns	Ns	2	2	II	-	A	A	A
Ando	53	53	53	53	Ns		1	1	Ⅱ	-	A	A	A
JCVI SC001	53	53	53	53	Ns		3	3	II	-	A	A	A
SJD4	53	53	53	53	X	X	1	1	Ⅱ	-	A	A	A
ATCC 49417	53	53	53	53	X	X	1	1	III	-	A	A	A
SJD5	53	53	53	53	X	X	3	3	I	-	B	B	B
15_9	53	53	53	53					IV	-	B	B	Ns
381OKJP	53	53	53	53					V	-	B	B	B
WW3039	53	X	53	53	A1		3	3	II	-	B	B	B
F0568	70A	70	70	70	A1		2	2	Ⅱ	-	A	A	A
W4087	70A	70	70	70	A1		2	2	Ⅱ	-	A	A	A
WW2885	70A	70	70	70	A1		2	2	I	-	A	A	A
F0185	70A	70	70	70	A1		2	2	II	-	A	A	A
381	70A	70	70	70	A1		4	4	Ⅰ	-	A	A	A
ATCC 33277	70A	70	70	70	A1		4	4	I	(M)	A	A	A
UBA8864	70A	70	70	70	A1		4	4	I	(M)	A	A	A
KCOM 2802	70A	70	70	70	A2	E	1	1	Ⅱ	-	A	A	A
A7436	70A	70	70	70	A2	E	1	1	IV	-	X	B	B
WW2842	70A	70	70	70	A2	E	2	2	I	-	A	A	A
**D83T3**	70A	70	70	70	A2	E	2	2	II	-	A	A	A
WW3102	70A	70	70	70	A2	E	2	2	II	-	A	A	A
WW5019	70A	70	70	70	A2	E	2	2	II	-	A	A	A
WW2096	70A	70	70	70	A2	E	2	2	III	-	A	A	A
CP3	70A	70	70	70	A2	E	2	2	IV	-	A	A	A
KCOM 2805	70A	70	70	70	A2	E	3	3	Ⅱ	-	A	A	A
KCOM 2804	70A	70	70	70	A2	E	3	3	II	-	A	A	A
KCOM 2799	70A	70	70	70	A2	E	3	3	III	-	B	B	B
HG66	70A	70	70	70	A2	E	4	4	I	-	A	A	A
KCOM 2797	70A	70	70	70	A2	E	4	4	II	-	A	A	A
H3	70A	70	70	70	A2	X	3	3	I	-	A	A	A
SU60	70A	70	70	70	B (M)	Ns	3	3	IV	-	B	B	B
WW5127	70A	70	70	70	C		1	1	Ⅳ	-	B	B	B
3A1	70A	70	70	70	C		4	4	Ⅱ	-	A	A	A
KCOM 2800	70A	70	70	70	C		4	4	Ⅱ	-	A	A	A
KCOM 2803	70A	70	70	70	C		4	4	II	-	A	A	A
F0566	70A	70	70	70	D		3	3	I	-	A	A	A
TDC 60	70A	70	70	70	D		4	4	Ⅱ	-	A	A	A
KCOM 3001	70A	70	70	70	D		4	4	II	-	A	A	A
3_3	70A	70	70	70	Ns	E	4	4	I	-	A	A	A
F0569	70A	70	70	70	Ns	Ns	4	4	II	-	A	A	A
F0570	70A	70	70	70	X	X	2	2	Ⅰ	-	A	A	A
**EM3**	70A	70	70	70	X	X	3	3	III	-	A	A	A
SJD11	70A	70	70	70	X	X	4	4	Ⅱ	-	A	A	A
SJD12	70A	70	70	70	X	X	4	4	I	-	A	A	A
WW2881	70A	70	70	70	X		3	3	I	-	A	A	A
13_1	70A	70	70	70					II	-	A	A	A
WW2952	70A	70	70	X	A2	E	2	2	Ⅱ	-	A	A	A
**TV14**	70A (M)	70	70	70	D		2	2	Ⅱ	-	A	A	A
MP4-504	70B	70	70	70	A2	E	2	2	Ⅰ	-	A	A	A
**Kyudai-3**	70B	70	70	70	A2	E	2	2	Ⅱ	-	A	A	A
**1436**	70B	70	70	70	A2		2	2	Ⅰ	-	A	A	A
**JKG9**	70B	70	70	70	A2		2	2	Ⅰ	-	A	A	A
**1439**	70B	70	70	70	A2		2	2	Ⅱ	-	A	A	A
**B42**	70B	70	70	70	A2		2	2	Ⅳ	-	A	A	A
W83	X	70	70	70	A2	E	1	1	Ⅳ	-	B	B	B
W50	X	70	70	70	A2	X	1	1	IV	-	B	B	B
**JKG10**	X	70	70	70	A2	X	4	4	Ⅱ	-	A	A	A
**B158**	X	70	70	70	A2		2	2	Ⅱ	-	A	A	A
**Kyudai-4**	X	70	70	70	A2		3	3	II	-	A	A	A
**222**	φ	φ	φ	φ	X	X	1	1	Ⅳ	-	B	B	B
WW3040	φ	φ	φ	φ	φ		2	2	Ⅱ	-	A	A	A

• Strains with bold-type are genome-sequenced in this study.

• Genotyping (I-V; A or B) in each gene is shown in text.

• The *fimB* gene cannot be classified because of high homogeneity.

• M, a possible point mutation, such as a nonsense mutation, was detected. However, as only one mutation had little influence, genetic analysis was still performed.

• X, a possible mutation, except for a point mutation, was detected. Although the corresponding genes were detected, genetic analyses were not performed.

• Ns, a sequence of Ns was detected in a gene because of complementation in the process of scaffolding in the NGS analysis. Although the corresponding genes were recognized, genetic analyses were not performed.

• φ, corresponding gene was not detected.

• Light blue box indicates the genes were detected in the same contig as *mfa1*. I.e., when *mfa1* to *mfa5-1*, *ragA* and *ragB* are light blue, but *mfa5-2* is white, then *mfa1* to *ragB* are successively arranged (but not *mfa5-2*).

• Gray indicates the genes were not detected in the same contig as *mfa1*.

• The *fimA* to *fimE* genes were arranged in sequence in all but two strains.

## Detection of the *mfa*, *rag*, and *fim* gene clusters

In addition to the 12 strains described above, the *mfa*, *rag*, and *fim* gene clusters were extracted from the genome data of 62 *P*. *gingivalis* strains published on eHOMD (Table 3). The *mfa1*, *mfa2*, *mfa3*, *mfa4*, *mfa*5, *ragA*, and *ragB* genes were tandemly arranged in this order when both the *mfa* and *rag* gene clusters were detected in a contig. Surprisingly, there were strains possessing two *mfa5* genes with a tandem arrangement. Hereafter, the first and second *mfa5* genes are called *mfa5-1* and *mfa5-2*, respectively. In the *fim* gene cluster, *fimA* to *fimE* were also arranged in sequence in all but two strains that contained the incomplete gene.

## Genetic diversity in the *mfa* gene cluster

**Fig 2:**
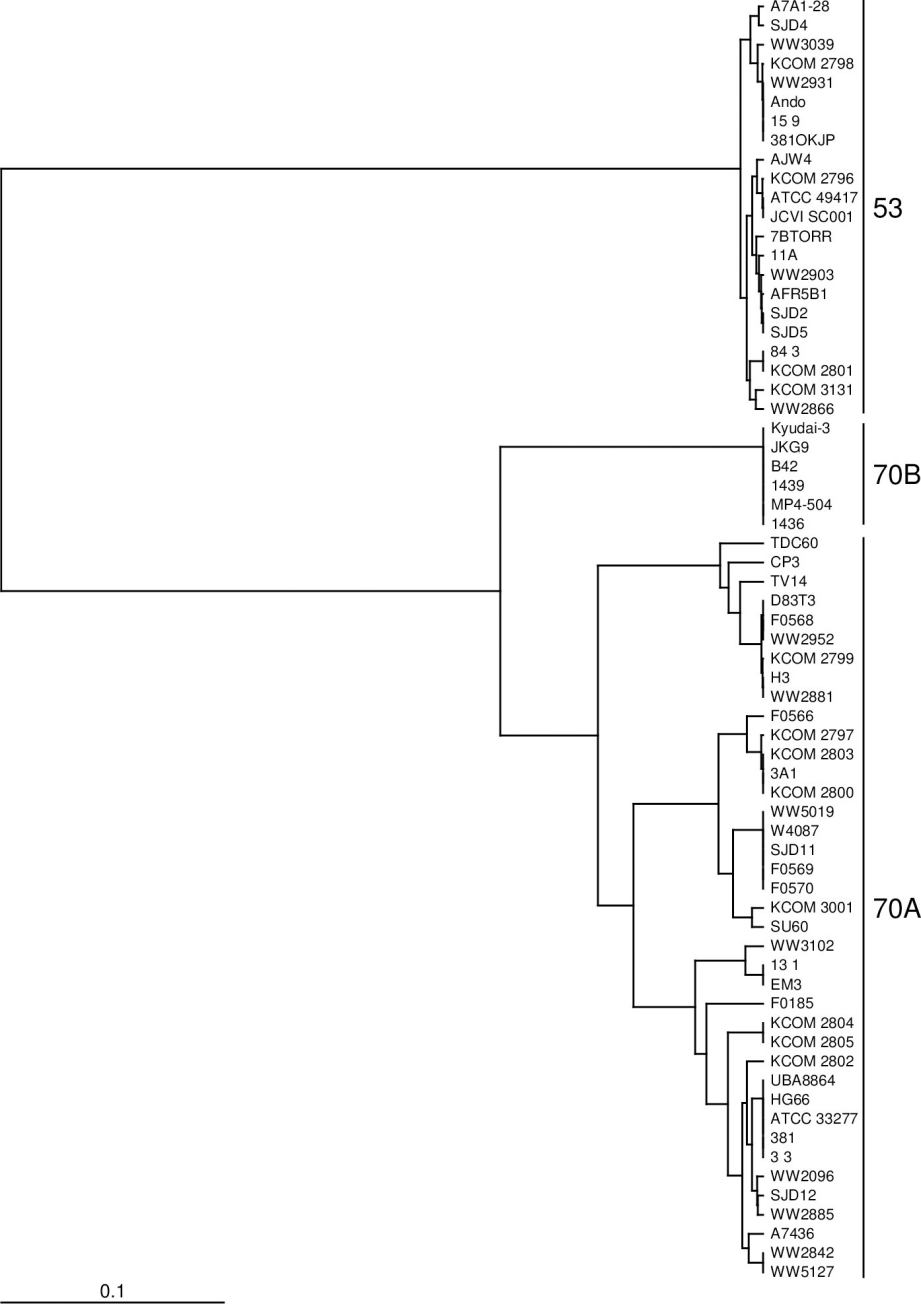
Phylogenetic tree of the *mfa1* gene. A phylogenetic tree was constructed with TreeView X through a multiple sequence alignment analysis using ClustalΩ. The *mfa1* gene is primarily classified into genotypes 53 and 70. Additionally, genotype 70 forms two clusters, called genotypes 70A and 70B.

Genetic diversity was phylogenetically analyzed using ClustalΩ. The *mfa1* gene was primarily classified into two genotypes, 53 and 70, as reported previously [20, 21] (Table 3 and Fig 2). Additionally, two cluster formations were observed in genotype 70. Here, we refer to them as genotypes 70A and 70B. The *mfa2*, *mfa3*, and *mfa4* genes were classified into two genotypes consistent with those of *mfa1*, although genotype 70 was not divided into subtypes (Table 3; S1–S3 Figs). Additionally, the phylogenetic distance of *mfa2* to *mfa4* was less than that of *mfa1*; in particular, *mfa2* showed high homogeneity among the strains.

**Fig 3:**
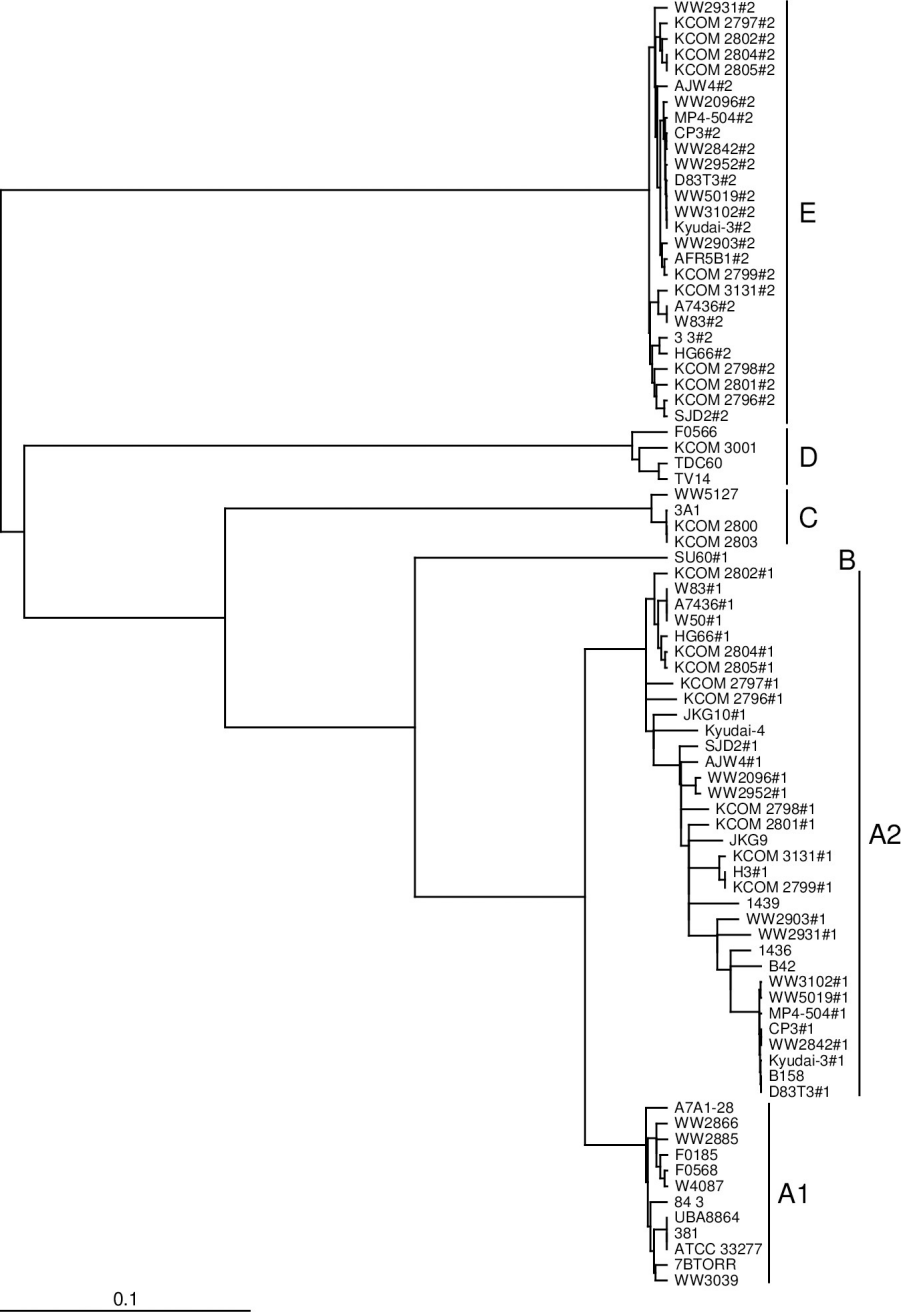
Phylogenetic tree of the *mfa5* gene. A phylogenetic tree was constructed with TreeView X through a multiple sequence alignment analysis using ClustalΩ. The *mfa5* gene is primarily classified into 5 genotypes of A, B, C, D, and E. Additionally, genotype A forms 2 clusters, called genotypes A1 and A2. Strain names appending with either #1 or #2 indicate the first and second *mfa5*, respectively.

The classification of *mfa5* was completely different from that of *mfa1* to *mfa4*. It was primarily classified into five genotypes, A to E (Table 3 and Fig 3). Additionally, genotype A showed two clusters, called genotypes A1 and A2. The gene length between the genotypes differed by more than double in some instances (Table 4): 2,946 to 3,747 in genotype A (including genotypes A1 and A2); 4,595 bp in genotype B; 5,049 bp in genotype C; 4,020 bp in genotype D; and 6,615 bp in genotype E (except for 6,614 of KCOM 2798 because of missing a nucleotide). Intriguingly, when 2 *mfa5* genes were detected, the first (*mfa5*-*1*) and second (*mfa5*-*2*) genotypes were exclusively genotypes A2 and E, respectively.

**Table 4:** The *mfa5* genotype and gene length.

Strain	Genotype	Gene length (bp)	Strain	Genotype	Gene length (bp)
Kyudai-4	A2	2946	F0566	D	4020
B42	A2	3087	KCOM_3001	D	4020
JKG9	A2	3096	TDC60	D	4020
1439	A2	3144	TV14	D	4020
1436	A2	3243	SU60#1	B (M)	4595
KCOM_2798#1	A2	3675	3A1	C	5049
A7436#1	A2	3678	KCOM_2800	C	5049
AJW4#1	A2	3678	KCOM_2803	C	5049
CP3#1	A2	3678	WW5127	C	5049
H3#1	A2	3678	KCOM_2798#2	E (M)	6614
HG66#1	A2	3678	KCOM_2799#2	E	6615
KCOM_2796#1	A2	3678	3_3#2	E	6615
KCOM_2797#1	A2	3678	A7436#2	E	6615
KCOM_2799#1	A2	3678	AFR5B1#2	E	6615
KCOM_2802#1	A2	3678	AJW4#2	E	6615
KCOM_2804#1	A2	3678	CP3#2	E	6615
KCOM_2805#1	A2	3678	D83T3#2	E	6615
KCOM_3131#1	A2	3678	HG66#2	E	6615
MP4-504#1	A2	3678	KCOM_2796#2	E	6615
SJD2#1	A2	3678	KCOM_2797#2	E	6615
WW2096#1	A2	3678	KCOM_2801#2	E	6615
WW2842#1	A2	3678	KCOM_2802#2	E	6615
WW2903#1	A2	3678	KCOM_2804#2	E	6615
WW2931#1	A2	3678	KCOM_2805#2	E	6615
WW2952#1	A2	3678	KCOM_3131#2	E	6615
WW3102#1	A2	3678	Kyudai-3#2	E	6615
WW5019#1	A2	3678	MP4-504#2	E	6615
W50#1	A2	3681	SJD2#2	E	6615
W83#1	A2	3681	W83#2	E	6615
A7A1-28	A1 (M)	3683	WW2096#2	E	6615
381	A1	3684	WW2842#2	E	6615
7BTORR	A1	3684	WW2903#2	E	6615
84_3	A1	3684	WW2931#2	E	6615
F0185	A1	3684	WW2952#2	E	6615
F0568	A1	3684	WW3102#2	E	6615
W4087	A1	3684	WW5019#2	E	6615
WW2866	A1	3684			
WW2885	A1	3684			
WW3039	A1	3684			
ATCC_33277	A1	3684			
UBA8864	A1	3687			
JKG10#1	A2	3744			
B158	A2	3747			
D83T3#1	A2	3747			
KCOM_2801#1	A2	3747			
Kyudai-3#1	A2	3747			

• Appendages #1 and #2 with strain names indicate the first and second mfa5 genes, respectively.

• M, a possible point mutation, such as a nonsense mutation, was detected. However, as only one mutation had little influence, genetic analysis was still performed.

### Protein structure and sequence analyses of Mfa1 to Mfa5

The protein structures of Mfa1 to Mfa5 between different genotypes were predicted and comparatively analyzed with SWISS-MODEL using the X-ray crystal structure data based on the gene information of ATCC 33277 as a template.

**Fig 4:**
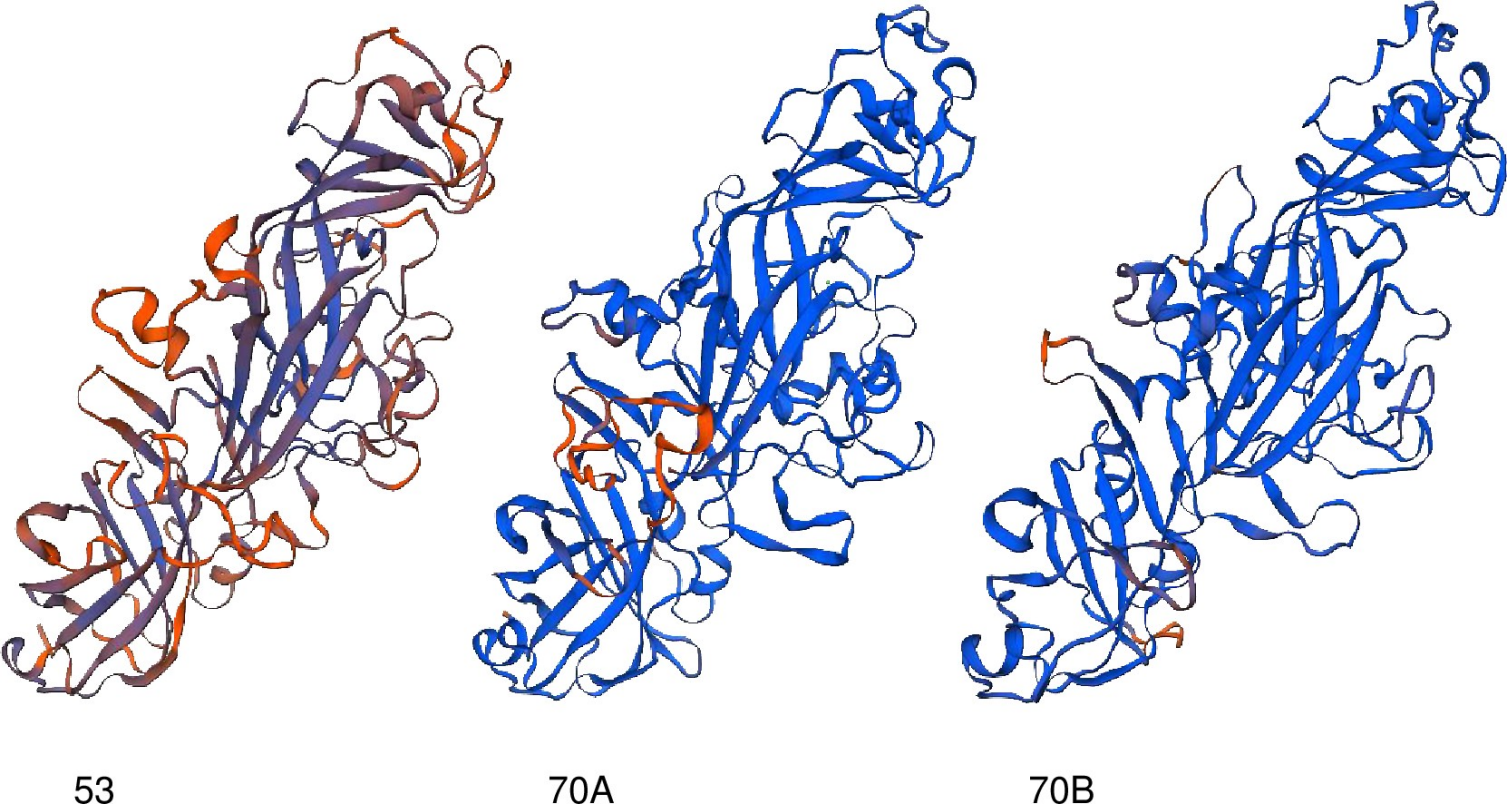
Protein structure homology-modeling of Mfa1. The mature form of the amino acid sequences of genotypes 53 (Ando), 70A (ATCC 33277), and 70B (JKG9) were subjected to SWISS-MODEL analysis. Homology modeling was performed using Mfa1 of ATCC 33277 (5nf3.1.A in PDB) as a template. The quality of protein structure models is indicated by qualitative model energy analysis (QMEAN): blue and red indicate good and bad specific feature quality scores, respectively.

In the Mfa1 protein maturation of ATCC 33277, precursor peptides undergo removal of Arg^49^ at the N-terminus during processing, including digestion with a signal peptidase and gingipain [46]. Therefore, we submitted the mature forms of the amino acid sequences deleting the N-terminal peptide corresponding to the Arg^49^ of respective genotypes of 53 (Ando), 70A (ATCC 33277), and 70B (JKG9) to SWISS-MODEL analysis. Mfa1 of genotype 70A naturally showed a high concordance rate (QMEAN value) in the overall structure (Fig 4). The structural model of genotype 70B also showed very high concordance. Genotype 53 had low QMEAN values overall, but the β-barrel structure, important for structure determination, and the donor-strand exchange mechanism was significantly conserved.

Mfa2 is not processed by gingipain [28, 43], and SignalP 5.0 predicted that a signal peptide before Ser^28^ was removed to yield the mature form. Mfa3 and Mfa4 are removed up to Arg^43^ and Arg^54^, respectively, by gingipain to yield the mature form [46]. These three protein structures of genotypes 53 (Ando) and 70 (ATCC 33277) showed a highly similar tertiary structure, as expected from the substantial homology of the primary structures (S4–S6 Figs).

**Fig 5:**
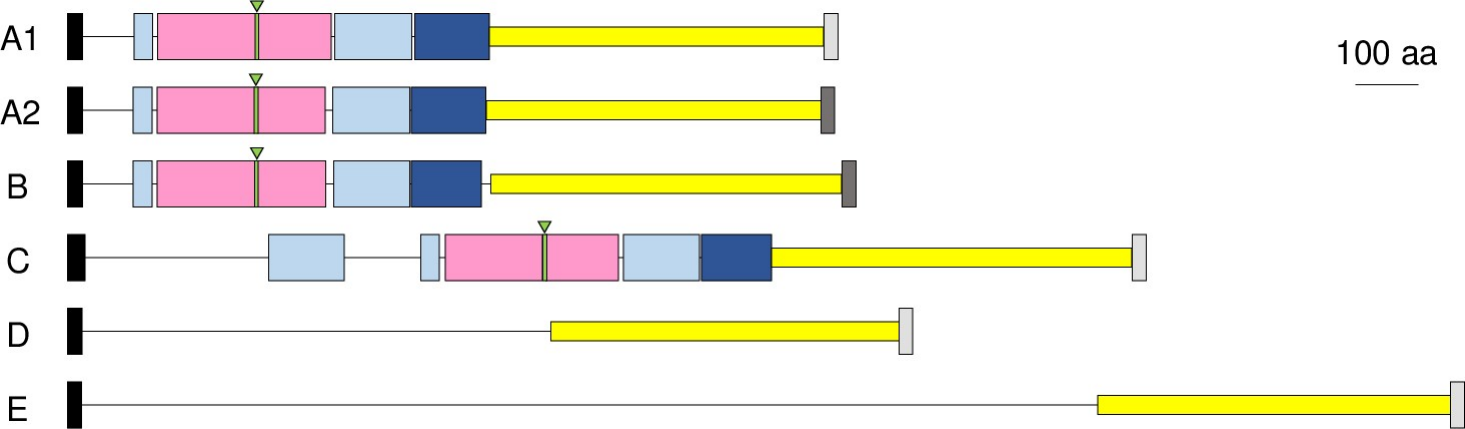
Domain arrangement of the Mfa5 protein in respective genotypes. Domain arrangements were designed based on the amino acid sequences deduced from the *mfa5* gene of the representative strain of each genotype. The N- to C-terminal of the sequence is shown from left to right. The black box on the far-left side is the predicted signal peptide. Light and dark blue are Ig-like domains, called D2 and D3 domains in the paper published by Heidler *et al*. [36]. Genotype C has an additional Ig-like domain. The pink is the von Willebrand factor A domain, green (with a triangle) is ARM2, and gray at the C-terminal is the CTD. The CTD was classified into two types: light (GAYVVSLQSPATSSNVRKVVVN) and dark (GAYIVHLQNAFTNDVHKVLVEY) gray. Yellow is a common region among all genotypes. A1, ATCC 33277; A2, the first *mfa5* (*mfa5-1*) of W83; B, the first *mfa5* (*mfa5-1*) of SU60; C, WW5127; D, TDC60; E, the second *mfa5* (*mfa5-2*) of W83.

In Mfa5, the primary structures were first analyzed (Fig 5). In all genotypes, the N-terminal signal peptide was predicted with high probability, and the CTD motif at the C-terminus was also conserved, though with two different sequence types (GAYVVSLQSPATSSNVRKVVVN and GAYIVHLQNAFTNDVHKVLVEY). Additionally, all genotypes contained homologous sequences in the C-terminal half. However, the VWF domain with the ARM2 loop and two Ig-like domains in the N-terminal half were detected only in four genotypes: A1, A2, B, and C. In these genotypes, the amino acid residues (Lys and Asn) involved in isopeptide bond formation were also located near the same positions in the four genotypes. Notably, an additional Ig-like domain was detected in genotype C. The tertiary structure of the N-terminal part, including VWF and 2 Ig-like domains, were highly conserved in genotypes A, B, and C (S7 Fig). Additionally, no similarity was detected in the N-terminal amino acid sequences between genotypes D and E; the N-terminal amino acid sequences of Mfa5 of TDC60 (genotype D) and the 2^nd^ Mfa5 of W83 (genotype E) showed only 10% identity. Furthermore, BLAST did not find homologous genes in the N-terminal half of genotypes D and E.

### Genetic diversity in the *rag* gene cluster, and its correlation to the *mfa* gene cluster

The *ragA* gene was classified into four genotypes (*ragA-1* to *ragA-4*), as reported previously [42] (Table 3 and S8 Fig). The classification of *ragB* completely matched that of *ragA*, as described previously [42] (Table 3 and S9 Fig). The *ragA*/*ragB* genes were present immediately downstream of the *mfa* gene cluster when the clusters were detected in the same contig. A relationship between the *mfa1* and *ragA*/*ragB* genotypes was observed: 20 strains with genotype-53 *mfa1* (detected for both of the *mfa1* and *ragA*/*ragB* genes) showed no detection of *ragA-4*, while detected *ragA-1*, *ragA-2*, and *ragA-3* in 8 (40%), 4 (20%), and 8 strains (40%), respectively. On the other hand, strains with 70A-genotype *mfa1* (38 strains) showed a low detection rate of *ragA-1* in 3 (7.9%), while substantially detected *ragA-2*, *ragA-3*, and *ragA-4* in 13 strains (34.2%), 8 strains (21%), and 14 strains (36.8%), respectively. In strains with the 70B genotypes of *mfa1*, all six strains correlated with *ragA-2*. No correlation was observed between the genotypes A1, A2, and E of *mfa5* with the *ragA*/*ragB* genotypes. However, although the sample number was small, genotypes C and D of *mfa5* correlated to *ragA-4* with high frequency: in three of four, and in two of four strains, respectively.

The *rag* gene cluster is followed by *rgpA*, which encodes gingipain in ATCC 33277 (Fig 1). However, the genes at this position were diverse among the strains, and other genes were detected, annotated as outer membrane proteins, lipoproteins, and hypothetical proteins. Additionally, no genetic diversity has been reported for *rgpA* [47].

### Genetic diversity in the *fim* gene cluster and its correlation with the *mfa* gene cluster

The *fimA* gene was classified as genotypes I to V, as previously reported (Table 3 and S10 Fig). The *fimB* gene showed an almost homogenous sequence (Table 3 and S11 Fig). The *fimC*, *fimD*, and *fimE* genes were divided into two genotypes with high consistency, respectively referred to as genotypes A and B (Table 3, and S12–S14 Figs). Genotype B was present in only 13/74 strains, respectively in *fimA* genotypes I (2/21), II (1/38), III (1/4), IV (7/9), and V (2/2) strains, thus showing a high incidence of genotype B in *fimA* genotypes IV and V. No correlation was found between either the *mfa1* and *fimA* genotypes, or the *mfa5* and *fimA* genotypes.

## Discussion

This study primarily examined the genetic diversity of the *mfa1* to *mfa5* genes encoding the Mfa1 fimbrial components of *P*. *gingivalis*. We first determined the *mfa1* genotype in the 12 strains of *P*. *gingivalis* in which the *mfa1* genotype had not been determined previously [20]. Of these, 11d out of 12 strains had genotype 70 of *mfa1*, and their *mfa1* gene sequences contained multiple mismatched bases compared to the primers used for PCR in the previous study, retrospectively indicating unsuccessful PCR. The remaining strain 222 likely misses *mfa1*. It is also evident now that the previous western blotting failed to detect the Mfa1 protein in strains B158, Kyudai-4, and TV14 likely due to nonsense mutation in *mfa1* preventing its expression [20]. Moreover, low protein expression and differential antigenicity might decrease the sensitivity of western blotting.

In addition to the above 12 strains, the genomic information of 62 strains of *P*. *gingivalis* published on the eHOMD site was used to analyze the genetic diversity in the *mfa*, *rag*, and *fim* gene clusters. We first examined the gene arrangement on the chromosome and found the *mfa* and *rag* gene clusters arranged consecutively when the genes in both clusters were all present in a contig. However, the genes downstream of *ragB* were diverse and appeared to be discontinuous. Additionally, the strains with the 2 *mfa5* genes often showed a defective assembly of the genes, possibly because there was a homologous sequence between the two *mfa5* genes (symbol X in Table 3). The *fimA* to *fimE* genes were detected in a cluster in all strains except the two strains.

Although the *mfa1* gene was primarily classified into genotypes 53 and 70 previously, we found the possibility that genotype 70 can be further divided into subtypes 70A and 70B (Fig 2). However, the protein structural modeling by SWISS-MODEL analysis showed a significantly high homology between genotypes 70A and 70B (Fig 4). The *mfa2*, *mfa3*, and *mfa4* genes were classified into two genotypes, which were consistent with the *mfa1* genotypes, although genotype 70 from *mfa2* to *mfa4* was not further divided. This suggests that *mfa1* to *mfa4* develop during synchronization. However, it should be noted that the differences between the genotypes of *mfa2* are very small, and the degree of diversity of *mfa3* and *mfa4* is less than that of *mfa1*. Furthermore, Mfa1 fimbriae were normally expressed even when *mfa1* was replaced with a different genotype [20]. Briefly, the *mfa1*-deficient mutant of ATCC 33277 (genotype 70A) restored Mfa1 fimbriae by introducing *mfa1* from Ando (genotype 53) *in trans*. This shows that *mfa2*, an anchor and length regulator [26], and both *mfa3* and *mfa4* are useful for fimbrial assembly [28–32] in different genotypes. The high conservation of the protein structures of Mfa1 to Mfa4 by SWISS-MODEL analysis also supports this result.

On the other hand, *mfa5* showed a classification that was completely different from that of the other *mfa* genes. It was classified into five genotypes from A to E, and genotype A was further divided into subtypes A1 and A2 (Fig 3). The genes also showed considerable differences in gene length between the different genotypes (Table 4). Surprisingly, there was a substantial number of strains holding two *mfa5* genes tandemly, and the first (*mfa5-1*) and second (*mfa5-2*) were exclusively genotypes A2 and E, respectively (Table 3). All genotypes showed the conserved C-terminal half and possessed CTD, indicating that Mfa5 is transported to the cell surface by T9SS, regardless of the genotype (Fig 5). However, N-terminals containing VWF domain, ARM2 loop, and the characteristic isopeptide bond were detected only in genotypes A1, A2, B, and C, whereas genotypes D and E did not have the N-terminal part. The binding site of the VWF domain in Mfa5 (of ATCC 33277, *mfa5*-genotype A1) was predicted to be blocked by an extended structure [36], and its function in the VWF domain remains unknown. On the other hand, it has been shown that a mutant strain expressing Mfa5 lacking the VWF domain reduced Mfa1-fimbrial expression, and the defective Mfa5 was no longer incorporated into the fimbrial structure [32]. However, Heidler *et al*. [36] indicated that a fatal mutation might occur in the overall structure of the defective Mfa5 lacking the VWF, and the function of the VWF domain alone might not be able to be examined. We are interested in the expression of Mfa1 fimbriae in a strain expressing Mfa5, encoded by the genotype-D *mfa5*. It would be also useful to examine fimbrial expression in a mutant strain deleted with genotype-A2 *mfa5* from a strain with both A2- and E-genotypes *mfa5*. Additionally, the SU60 strain possesses *mfa5* with genotype B and seems to have the secondary *mfa5* (*mfa5-2*), although the sequences have not been fully read. Therefore, the genotype of *mfa5-2* still needs to be determined. Heidler *et al*. [36] claimed that the *mfa5* gene might be transferred from streptococci because of the homology between the Ma5 and streptococcal adhesins (RrgA and GBS104); however, this finding could not explain the genetic diversity of the *mfa5*. It also could not explain why all genotypes of *mfa5* had the CTD found only in the phylum Bacteroidetes. Furthermore, it has been reported that Mfa1 fimbriae recognize SspB expressed on the cell surface of *Streptococcus gordonii*, which is the predominant bacterium in oral biofilms [48]. However, this examination was performed only using ATCC 33277, and should therefore be confirmed using strains expressing Mfa1 fimbriae composed of the proteins encoded by different genotypes of *mfa1* to *mfa5*.d.

The *ragA*/*ragB* genes were classified into four genotypes, as previously reported, and both genotypes were completely in agreement. An association between the genotypes of *ragA*/*ragB* and *mfa1* was observed; The *ragA-4* genotype was absent in strains with genotype-53 *mfa1*, and *ragA-1* rarely detected in strains with genotype-70A *mfa1*. Additionally, all six strains with genotype-70B *mfa1* showed *ragA-2*. Thus, *ragA*-4 was only detected in strains with the genotype-70A *mfa1*. It is not surprising that the genotypes of the *mfa1* and *raga*/*ragB* genes showed a relationship as both gene clusters were always tandemly arranged; however, the genetic diversity of *mfa5* between them showed no relationship.

The *fimA* was classified into genotypes I to V, as previously reported. Additionally, to our knowledge this is the first report of 2 genotypes in *fimC* to *fimE*. We also showed that *fimB* was highly conserved in *P*. *gingivalis*, similar to *mfa2*, both of which have similar functions. The majority of *fimC* to *fimE* were classified as genotype A, while genotype B was detected in *fimA*-genotypes IV and V with high frequency, suggesting that there is a relationship between the genotypes of *fimA*, *fimC*, *fimD*, and *fimE*. Given the high pathogenicity of the *fimA*-genotype IV [8, 15, 16], we are interested in the association between genotype B in *fimC* to *fimE*, and bacterial pathogenicity. However, no association was found between the *mfa1* and *fimA* genotypes or the *mfa5* and *fimA* genotypes.

## Conclusions

The *mfa1* gene was classified into two genotypes, 53 and 70, although genotype 70 could be further divided into two subtypes. The genotypes of *mfa2*, *mfa3*, and *mfa4* were consistent with those of *mfa1*. The classification of *mfa5* was independent and *mfa5* was classified into five genotypes and two subtypes. Surprisingly, there were strains with two *mfa5* genes. All *mfa5* genes have a common C-terminal part, including CTD, but not always VWF in the N-terminal portion. There seems to be a relationship between the *mfa1* genotype and the *ragA*/*ragB* genotypes, but not with the *fimA* genotype. Future studies should focus on the association between the genotypes of accessory proteins, such as Mfa5, and pathogenicity in *P*. *gingivalis*.

## Supporting information

S1 Fig Phylogenetic tree of the *mfa2* gene. A phylogenetic tree was constructed with TreeView X through a multiple sequence alignment analysis using ClustalΩ. The *mfa2* gene is primarily classified into genotypes 53 and 70.
(TIF)

S2 Fig Phylogenetic tree of the *mfa3* gene. A phylogenetic tree was constructed with TreeView X through a multiple sequence alignment analysis using ClustalΩ. The *mfa3 gene* is primarily classified into genotypes 53 and 70.
(TIF)

S3 Fig Phylogenetic tree of the *mfa4* gene. A phylogenetic tree was constructed with TreeView X through a multiple sequence alignment analysis using ClustalΩ. The *mfa4* gene is primarily classified into genotypes 53 and 70.
(TIF)

S4 Fig Protein structure homology-modeling of Mfa2. The mature form of the amino acid sequences of genotypes 53 (Ando) and 70 (ATCC 33277) were subjected to SWISS-MODEL analysis. Homology modeling was performed using the Mfa2 of ATCC 33277 (5nfi.1.A in PDB) as a template. The quality of protein structure models is indicated by qualitative model energy analysis (QMEAN): blue and red indicate good and bad quality specific feature scores, respectively.
(TIF)

S5 Fig Protein structure homology-modeling of Mfa3. The mature form of the amino acid sequences of genotypes 53 (Ando) and 70 (ATCC 33277) were subjected to SWISS-MODEL analysis. Homology modeling was performed using Mfa3 of ATCC 33277 (5nf4.1.A in PDB) as a template. The quality of protein structure models is indicated by qualitative model energy analysis (QMEAN): blue and red indicate good and bad quality specific feature scores, respectively.
(TIF)

S6 Fig Protein structure homology-modeling of Mfa4. The mature form of the amino acid sequences of genotypes 53 (Ando) and 70 (ATCC 33277) were subjected to SWISS-MODEL analysis. Homology modeling was performed using the Mfa4 of ATCC 33277 (4rdb.1.A in PDB) as a template. The quality of protein structure models is indicated by qualitative model energy analysis (QMEAN): blue and red indicate good and bad quality specific feature scores, respectively.
(TIF)

S7 Fig Protein structure homology-modeling of Mfa5. The mature form of the amino acid sequences of genotypes A1 (ATCC 33277), B (SU60), and C (WW5127) were subjected to SWISS-MODEL analysis. Homology modeling was computed using Mfa4 of ATCC 33277 (6to1.1.A in PDB) as a template. The quality of protein structure models is indicated by qualitative model energy analysis (QMEAN): blue and red indicate good and bad quality specific feature scores, respectively. There is a possible missing nucleotide or misreading in *mfa5-1* of SU60. This strain is the only genotype B. To add genotype B to this analysis, the sequence was modified with reference to the sequence of ATCC 3377 (“T” added between the 277^th^ and 278^th^ DNA).
(TIF)

S8 Fig Phylogenetic tree of the *ragA* gene. A phylogenetic tree was constructed with TreeView X through a multiple sequence alignment analysis using ClustalΩ. The *ragA* gene is classified into genotypes 1–4.
(TIF)

S9 Fig Phylogenetic tree of the *ragB* gene. A phylogenetic tree was constructed with TreeView X through a multiple sequence alignment analysis using ClustalΩ. The *ragB* gene is classified into genotypes 1–4.
(TIF)

S10 Fig Phylogenetic tree of the *fimA* gene. A phylogenetic tree was constructed with TreeView X through a multiple sequence alignment analysis using ClustalΩ. The *fimA* gene was classified into genotypes I–V.
(TIF)

S11 Fig Phylogenetic tree of the *fimB* gene. A phylogenetic tree was constructed with TreeView X through a multiple sequence alignment analysis using ClustalΩ. The *fimB* gene showed a homogeneous cluster.
(TIF)

S12 Fig Phylogenetic tree of the *fimC* gene. A phylogenetic tree was constructed with TreeView X through a multiple sequence alignment analysis using ClustalΩ. The *fimC* gene is classified into genotypes A and B.
(TIF)

S13 Fig Phylogenetic tree of the *fimD* gene. A phylogenetic tree was constructed with TreeView X through a multiple sequence alignment analysis using ClustalΩ. The *fimD* gene is classified into genotypes A and B.
(TIF)

S14 Fig Phylogenetic tree of the *fimE* gene. A phylogenetic tree was constructed with TreeView X through a multiple sequence alignment analysis using ClustalΩ. The *fimE* gene was classified into genotypes A and B.
(TIF)
