# Supplementary figures and images for: Diversity analysis of genes encoding Mfa1 fimbrial components in Porphyromonas gingivalis strains
Source: PLoS One. 2021 Jul 26;16(7):e0255111. doi: 10.1371/journal.pone.0255111 (PMC8313007; doi:10.1371/journal.pone.0255111)

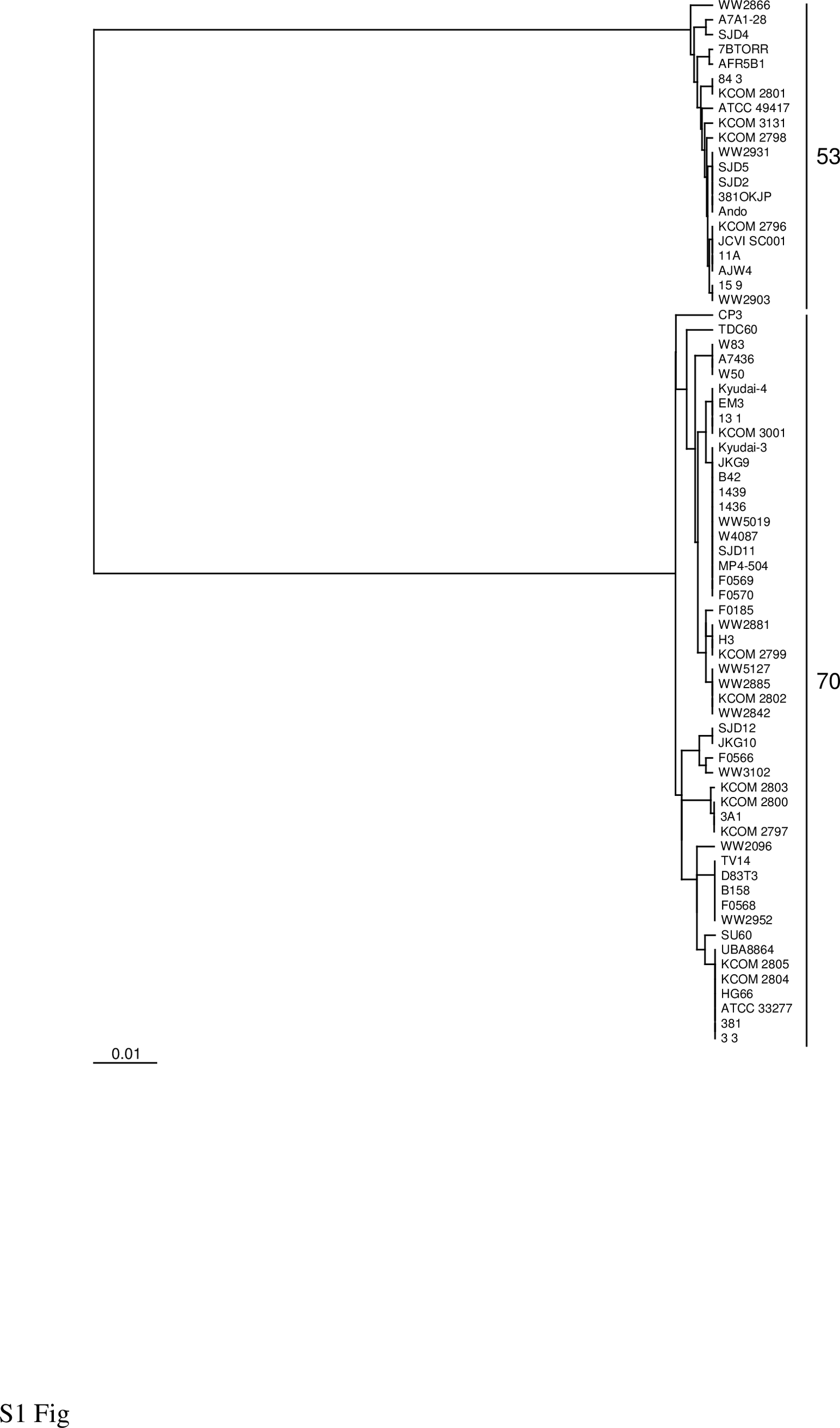

Supplement: S1 Fig — A phylogenetic tree was constructed with TreeView X through a multiple sequence alignment analysis using ClustalΩ. The mfa2 gene is primarily classified into genotypes 53 and 70. (TIF) [file pone.0255111.s001.tif]

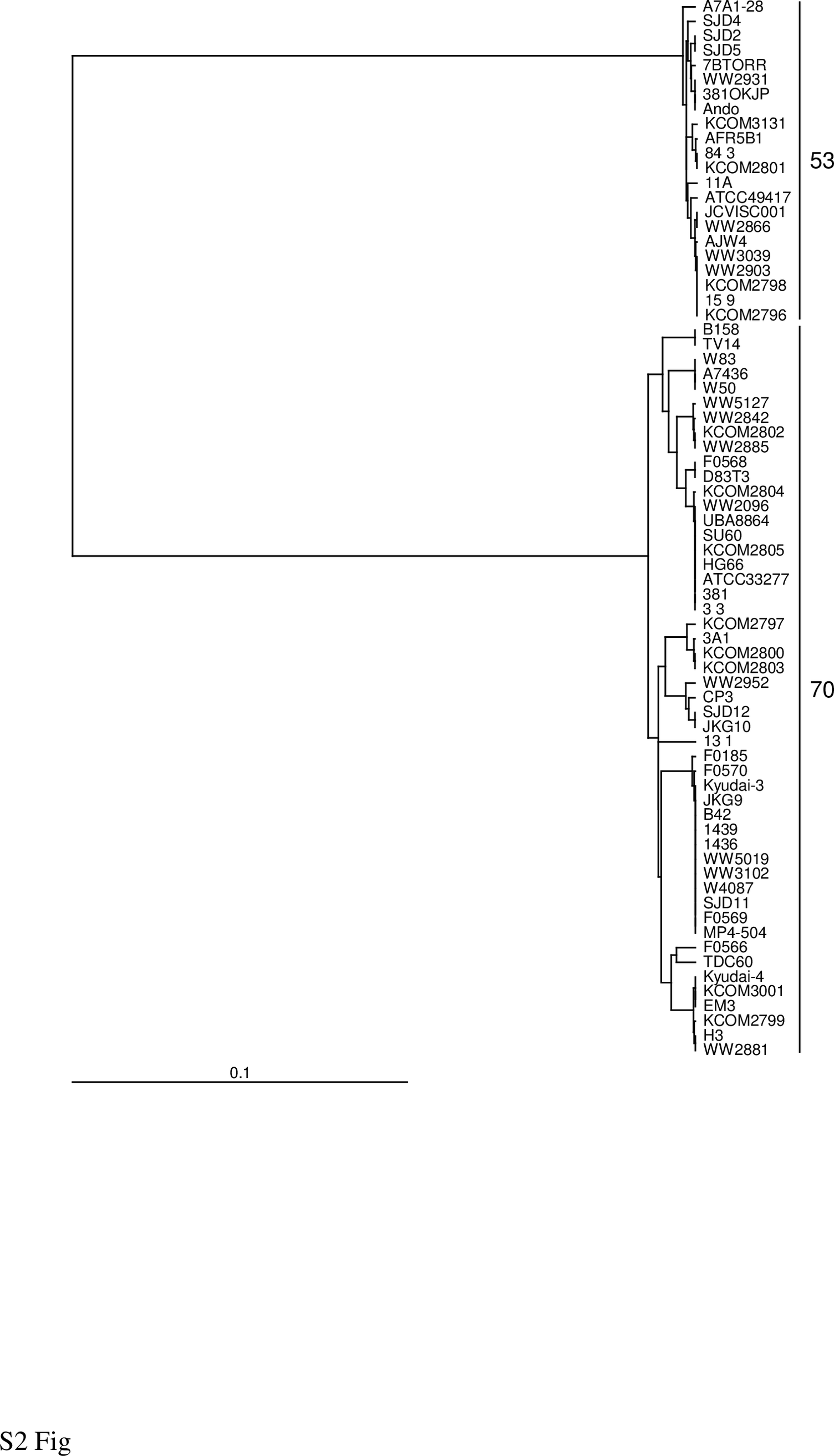

Supplement: S2 Fig — A phylogenetic tree was constructed with TreeView X through a multiple sequence alignment analysis using ClustalΩ. The mfa3 gene is primarily classified into genotypes 53 and 70. (TIF) [file pone.0255111.s002.tif]

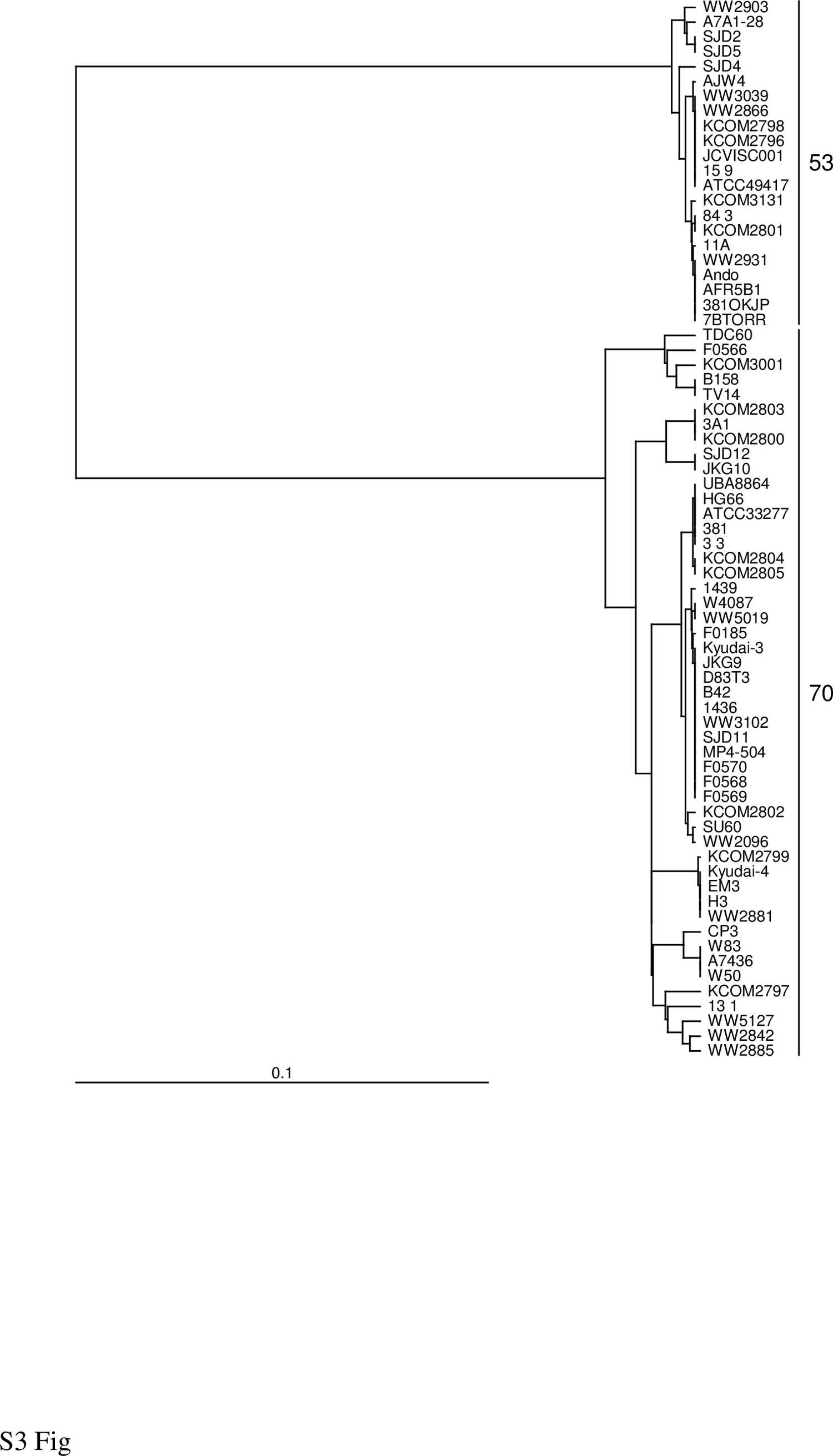

Supplement: S3 Fig — A phylogenetic tree was constructed with TreeView X through a multiple sequence alignment analysis using ClustalΩ. The mfa4 gene is primarily classified into genotypes 53 and 70. (TIF) [file pone.0255111.s003.tif]

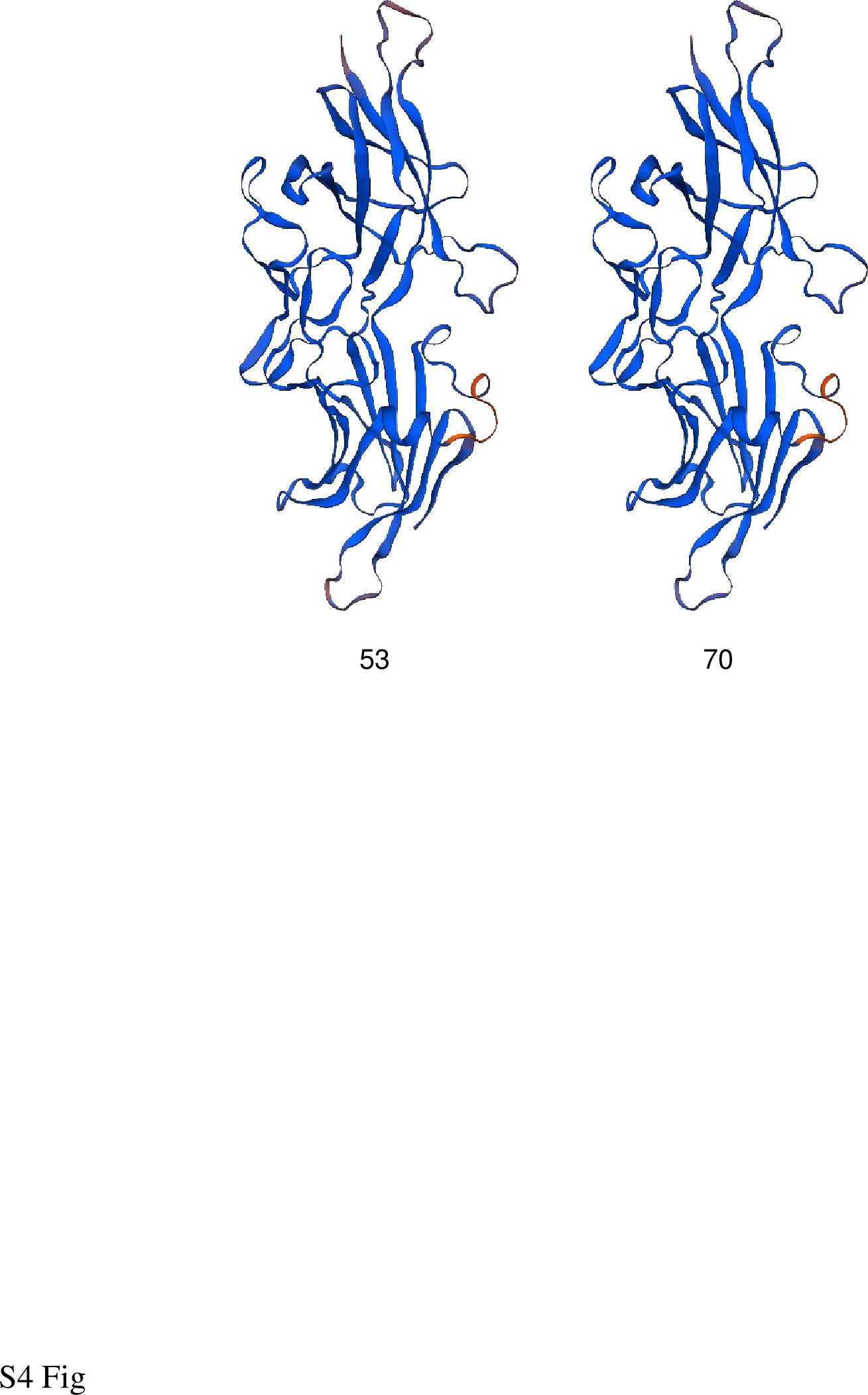

Supplement: S4 Fig — The mature form of the amino acid sequences of genotypes 53 (Ando) and 70 (ATCC 33277) were subjected to SWISS-MODEL analysis. Homology modeling was performed using the Mfa2 of ATCC 33277 (5nfi.1.A in PDB) as a template. The quality of protein structure models is indicated by qualitative model energy analysis (QMEAN): blue and red indicate good and bad quality specific feature scores, respectively. (TIF) [file pone.0255111.s004.tif]

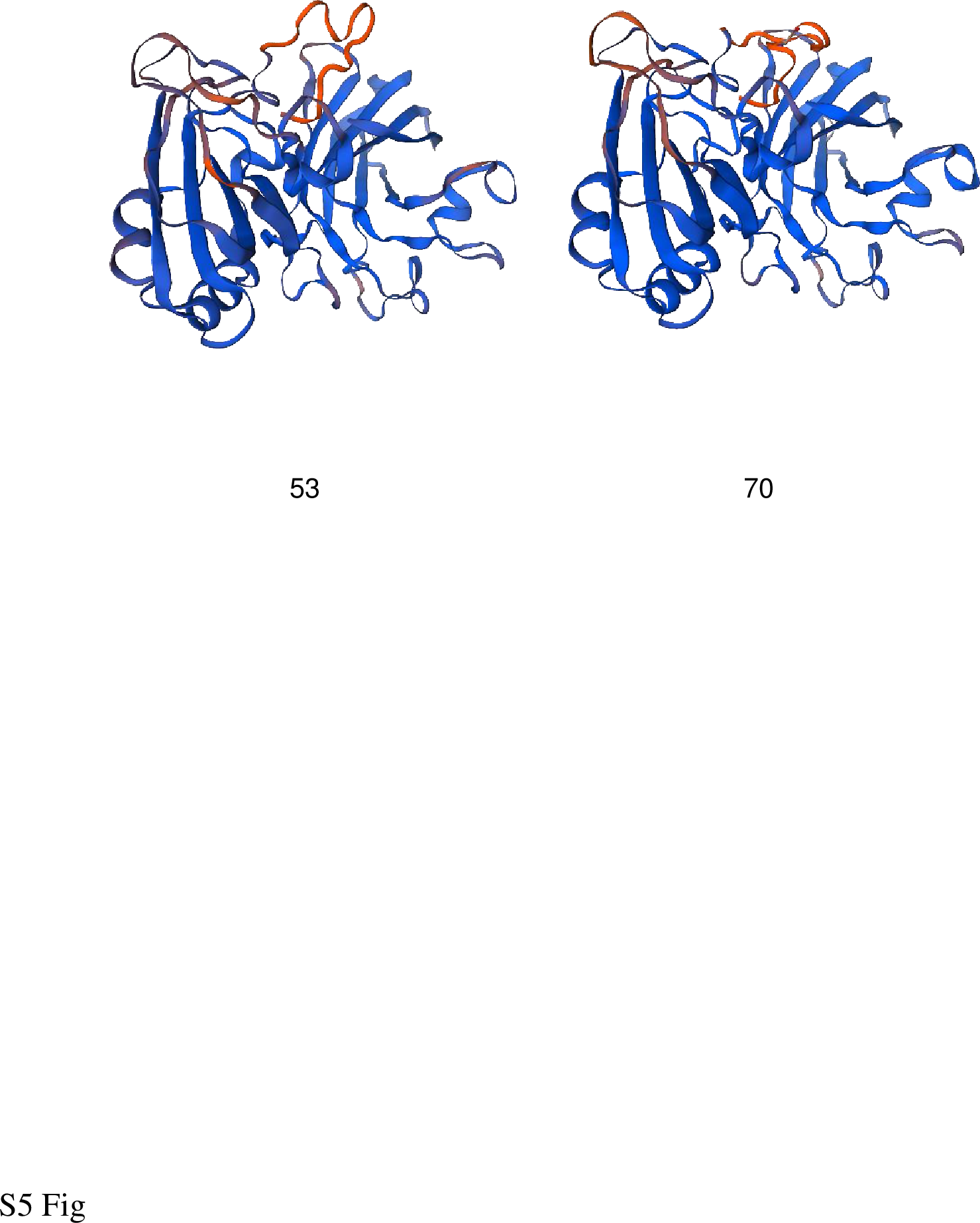

Supplement: S5 Fig — The mature form of the amino acid sequences of genotypes 53 (Ando) and 70 (ATCC 33277) were subjected to SWISS-MODEL analysis. Homology modeling was performed using Mfa3 of ATCC 33277 (5nf4.1.A in PDB) as a template. The quality of protein structure models is indicated by qualitative model energy analysis (QMEAN): blue and red indicate good and bad quality specific feature scores, respectively. (TIF) [file pone.0255111.s005.tif]

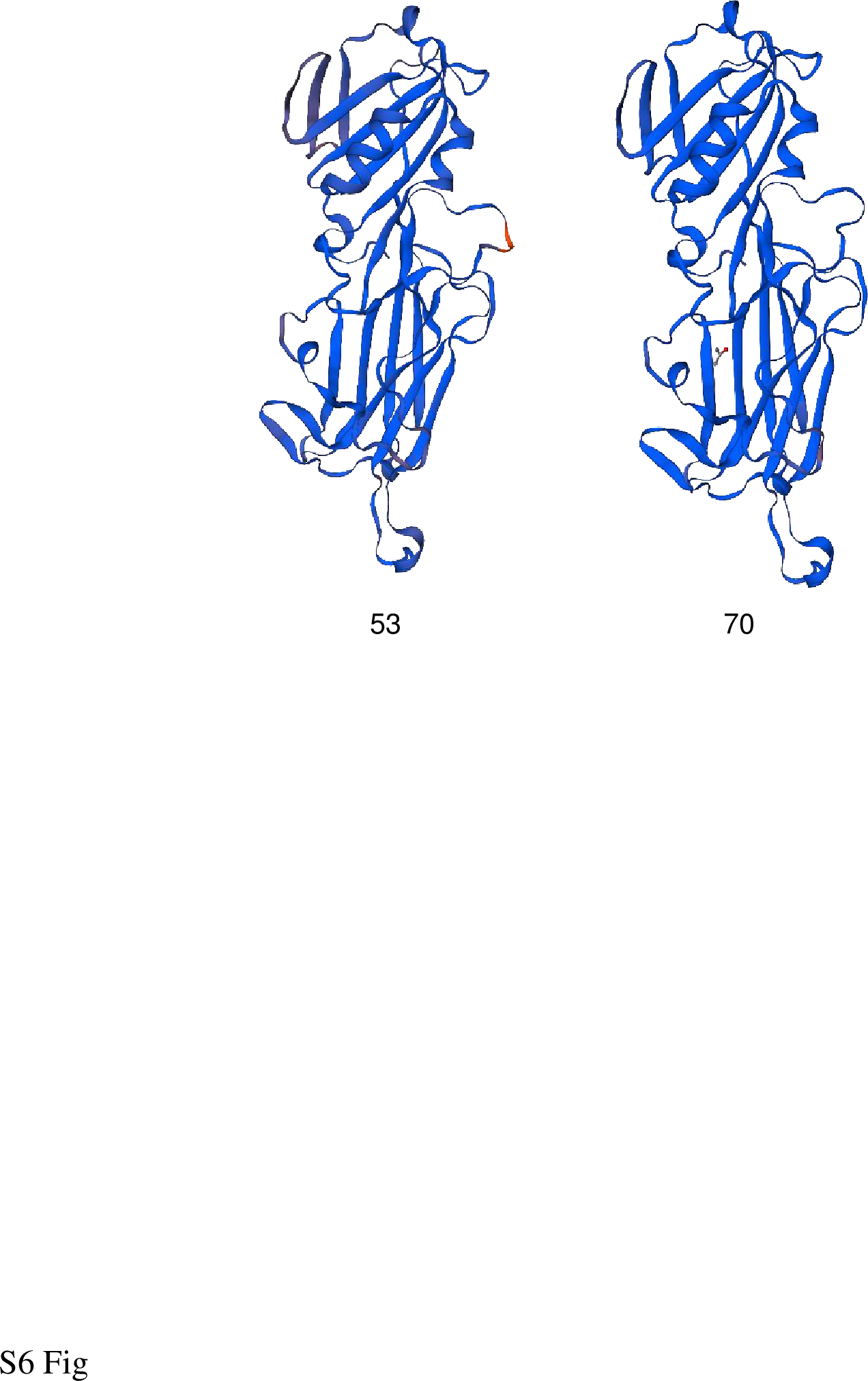

Supplement: S6 Fig — The mature form of the amino acid sequences of genotypes 53 (Ando) and 70 (ATCC 33277) were subjected to SWISS-MODEL analysis. Homology modeling was performed using the Mfa4 of ATCC 33277 (4rdb.1.A in PDB) as a template. The quality of protein structure models is indicated by qualitative model energy analysis (QMEAN): blue and red indicate good and bad quality specific feature scores, respectively. (TIF) [file pone.0255111.s006.tif]

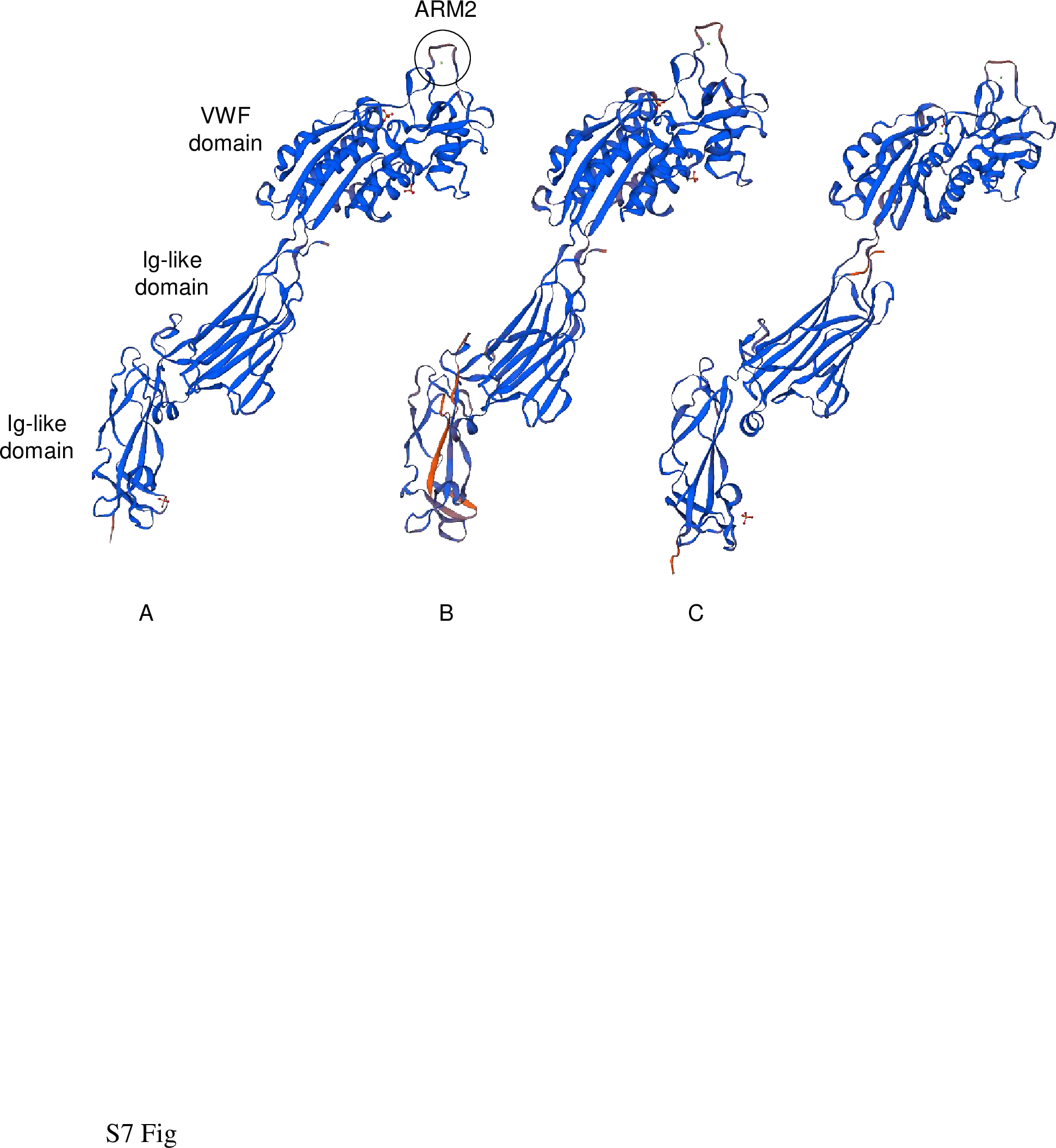

Supplement: S7 Fig — The mature form of the amino acid sequences of genotypes A1 (ATCC 33277), B (SU60), and C (WW5127) were subjected to SWISS-MODEL analysis. Homology modeling was computed using Mfa4 of ATCC 33277 (6to1.1.A in PDB) as a template. The quality of protein structure models is indicated by qualitative model energy analysis (QMEAN): blue and red indicate good and bad quality specific feature scores, respectively. There is a possible missing nucleotide or misreading in mfa5-1 of SU60. This strain is the only genotype B. To add genotype B to this analysis, the sequence was modified with reference to the sequence of ATCC 3377 (“T” added between the 277th and 278th DNA). (TIF) [file pone.0255111.s007.tif]

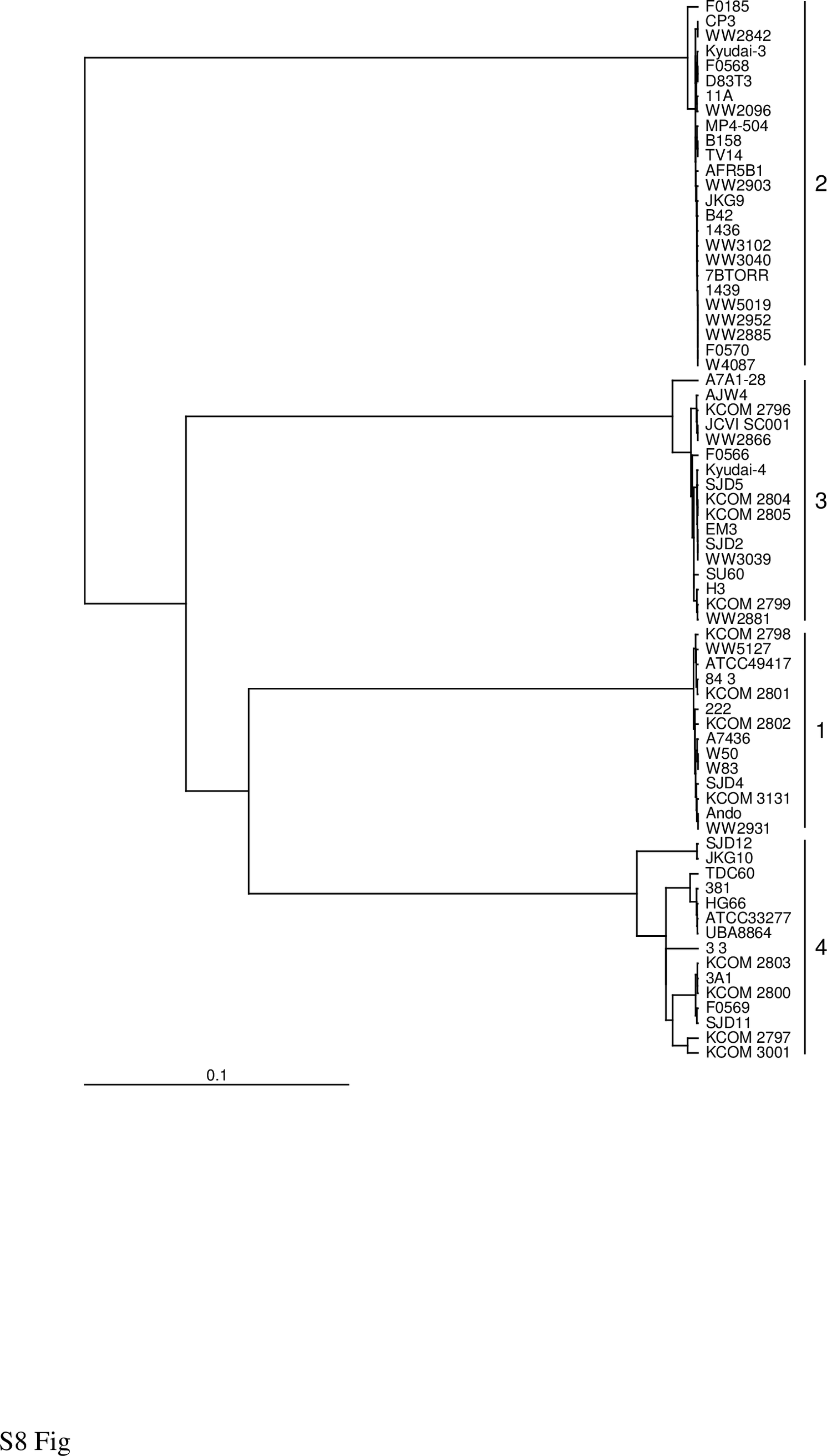

Supplement: S8 Fig — A phylogenetic tree was constructed with TreeView X through a multiple sequence alignment analysis using ClustalΩ. The ragA gene is classified into genotypes 1–4. (TIF) [file pone.0255111.s008.tif]

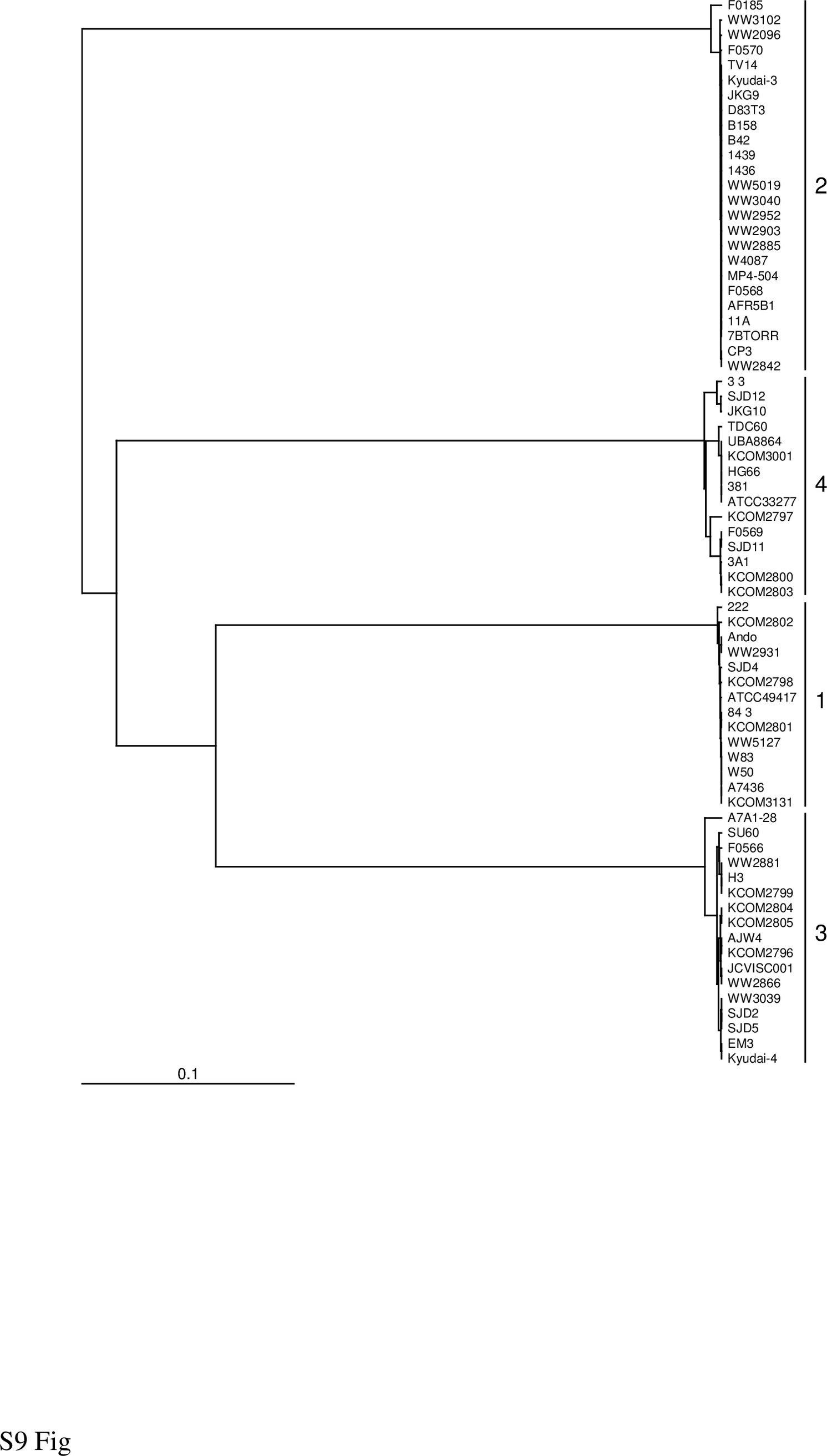

Supplement: S9 Fig — A phylogenetic tree was constructed with TreeView X through a multiple sequence alignment analysis using ClustalΩ. The ragB gene is classified into genotypes 1–4. (TIF) [file pone.0255111.s009.tif]

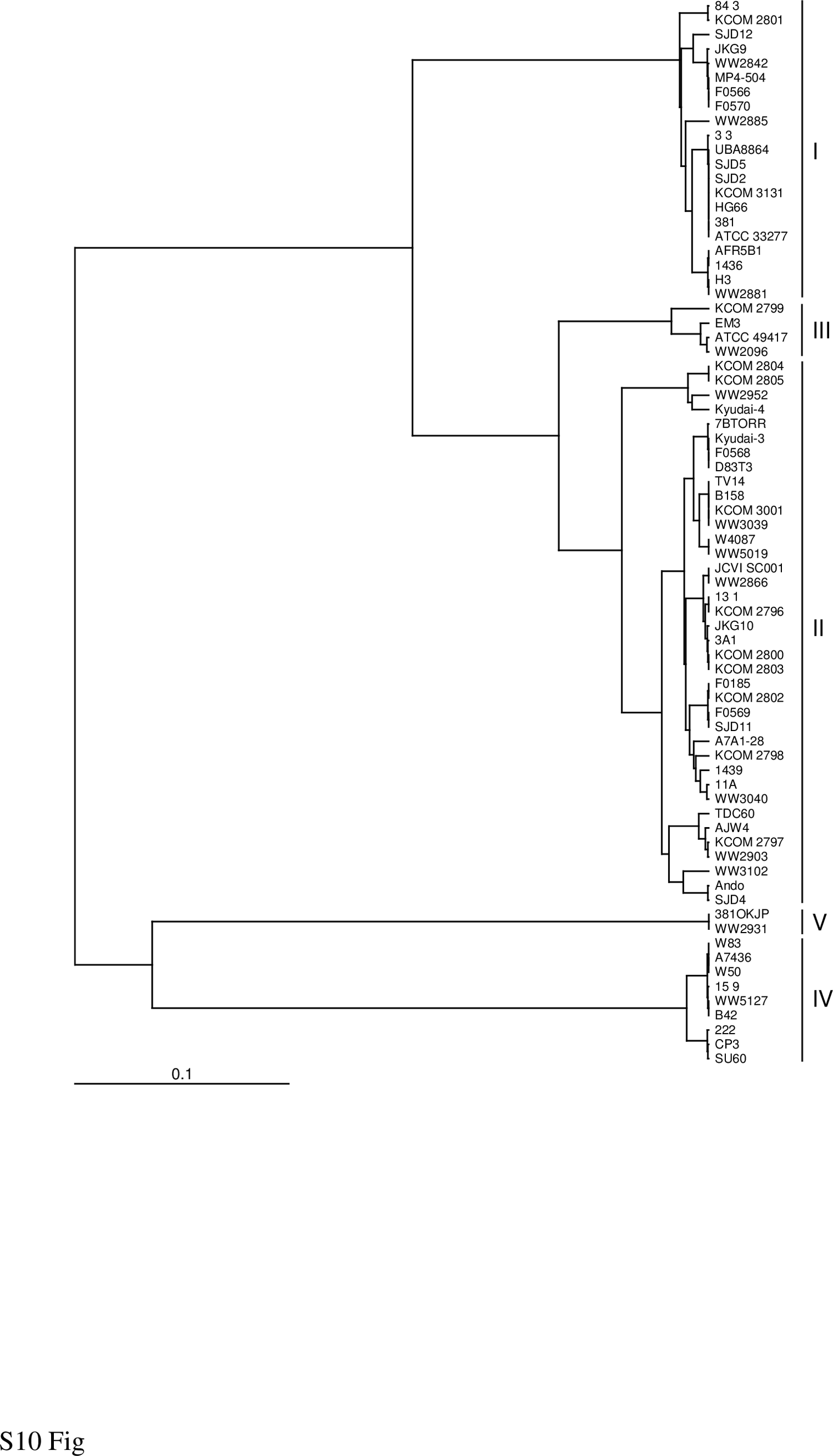

Supplement: S10 Fig — A phylogenetic tree was constructed with TreeView X through a multiple sequence alignment analysis using ClustalΩ. The fimA gene was classified into genotypes I–V. (TIF) [file pone.0255111.s010.tif]

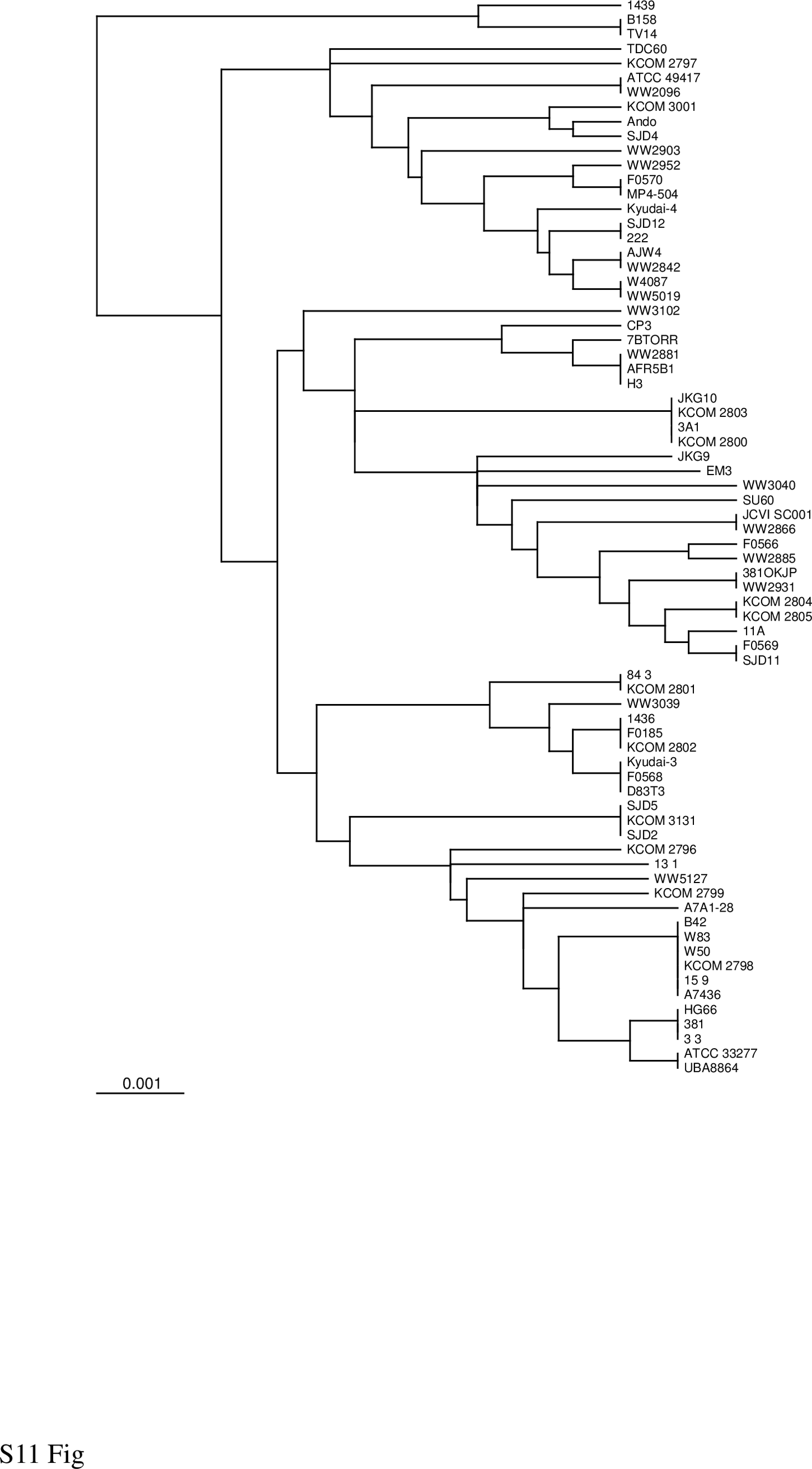

Supplement: S11 Fig — A phylogenetic tree was constructed with TreeView X through a multiple sequence alignment analysis using ClustalΩ. The fimB gene showed a homogeneous cluster. (TIF) [file pone.0255111.s011.tif]

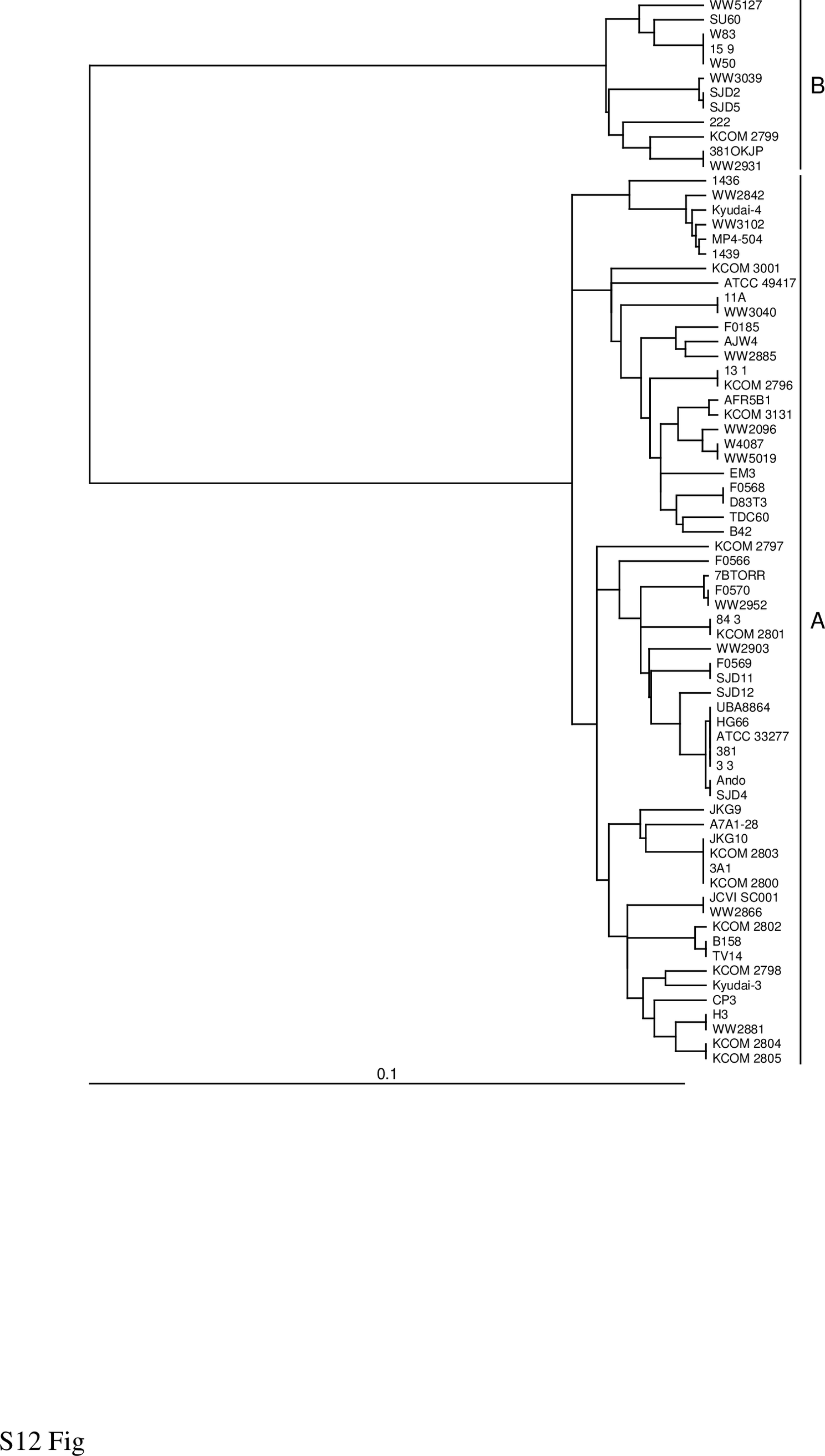

Supplement: S12 Fig — A phylogenetic tree was constructed with TreeView X through a multiple sequence alignment analysis using ClustalΩ. The fimC gene is classified into genotypes A and B. (TIF) [file pone.0255111.s012.tif]

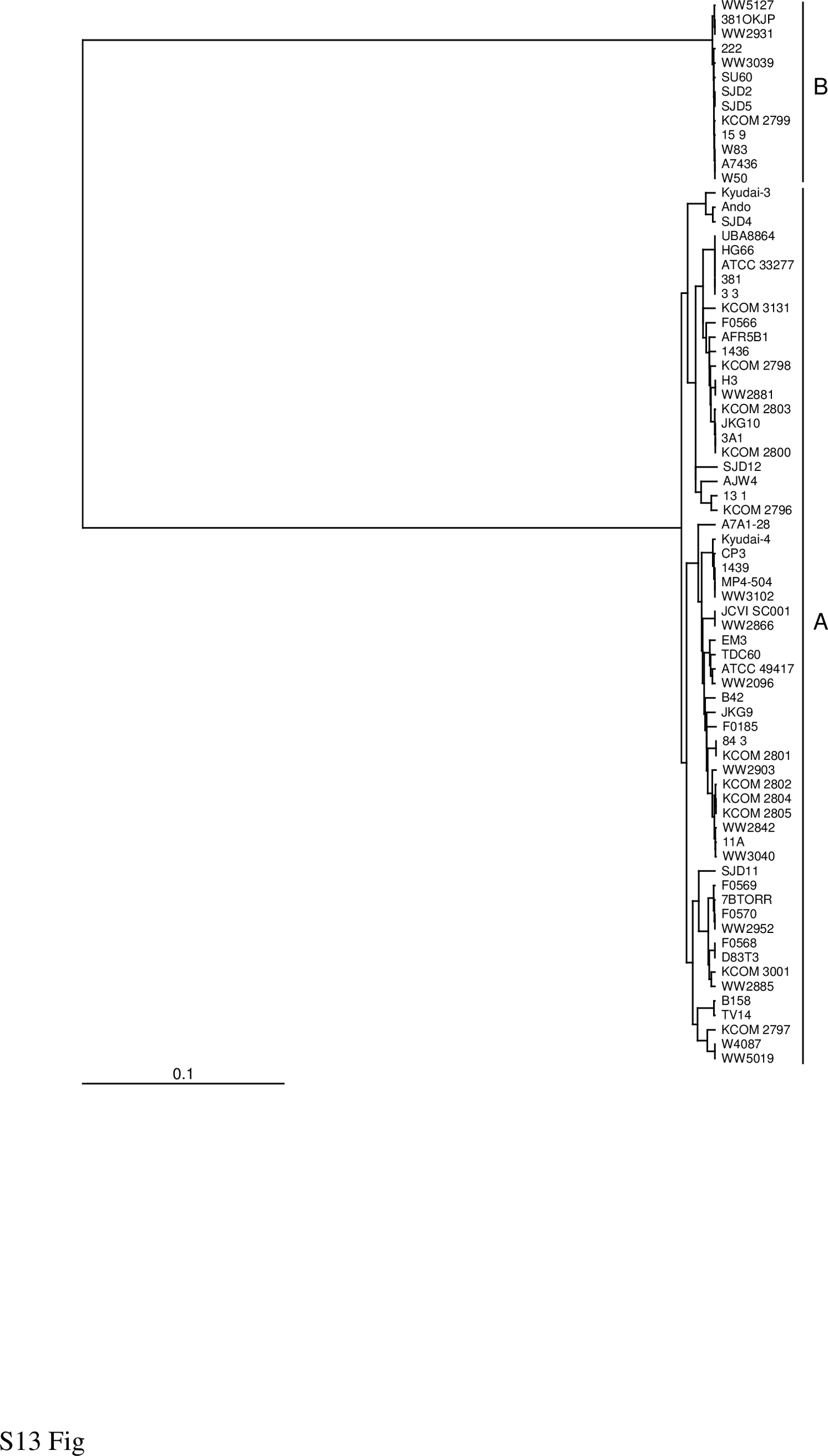

Supplement: S13 Fig — A phylogenetic tree was constructed with TreeView X through a multiple sequence alignment analysis using ClustalΩ. The fimD gene is classified into genotypes A and B. (TIF) [file pone.0255111.s013.tif]

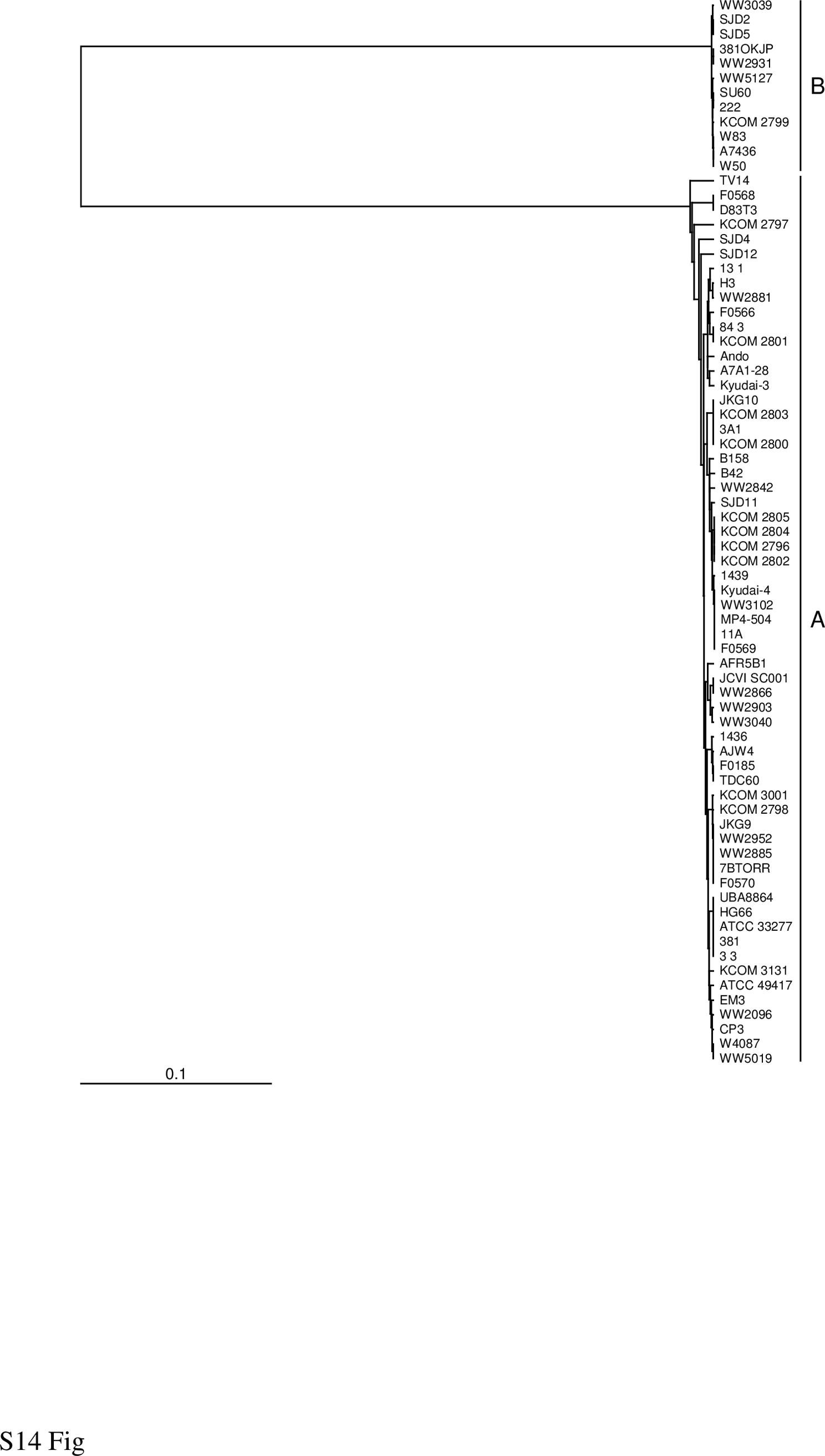

Supplement: S14 Fig — A phylogenetic tree was constructed with TreeView X through a multiple sequence alignment analysis using ClustalΩ. The fimE gene was classified into genotypes A and B. (TIF) [file pone.0255111.s014.tif]
